# Supplementary material for: Validation of the Perception Neuron system for full-body motion capture
Source: PLoS One. 2022 Jan 21;17(1):e0262730. doi: 10.1371/journal.pone.0262730 (PMC8782534; doi:10.1371/journal.pone.0262730)
Supplement: S1 Appendix — (PDF) [file pone.0262730.s001.pdf]

Appendix A: Bland Altman Plots

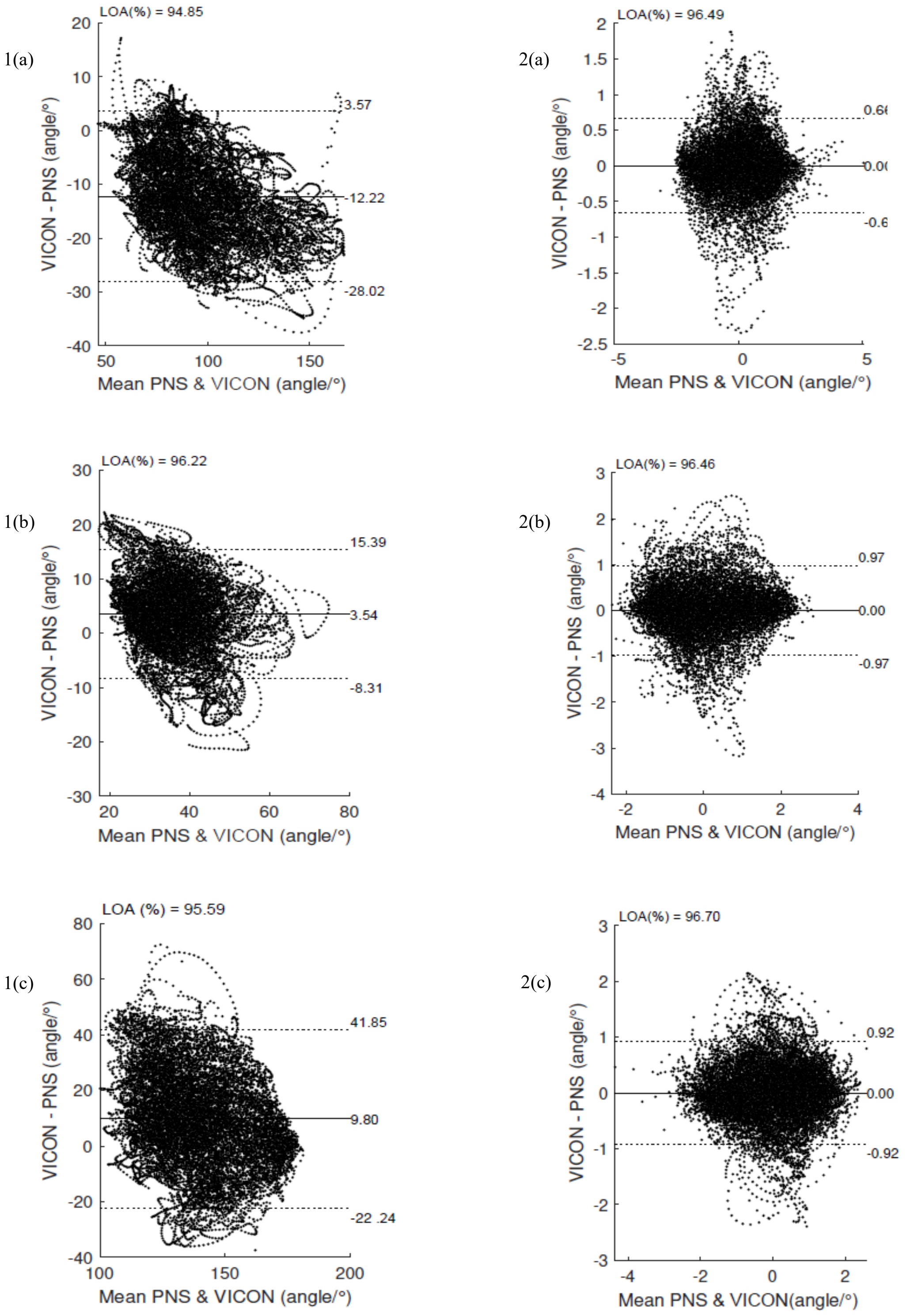

Bland-Altman plots of agreement for (a) elbow flexion/extension, (b) shoulder flexion/extension, (c) shoulder abduction/adduction for distance jog using (1) raw joint angles and (2) normalized joint angles. Solid horizontal lines represent the mean difference and the dashed horizontal lines represents the 95% limits of agreement (± 1.96 SD).

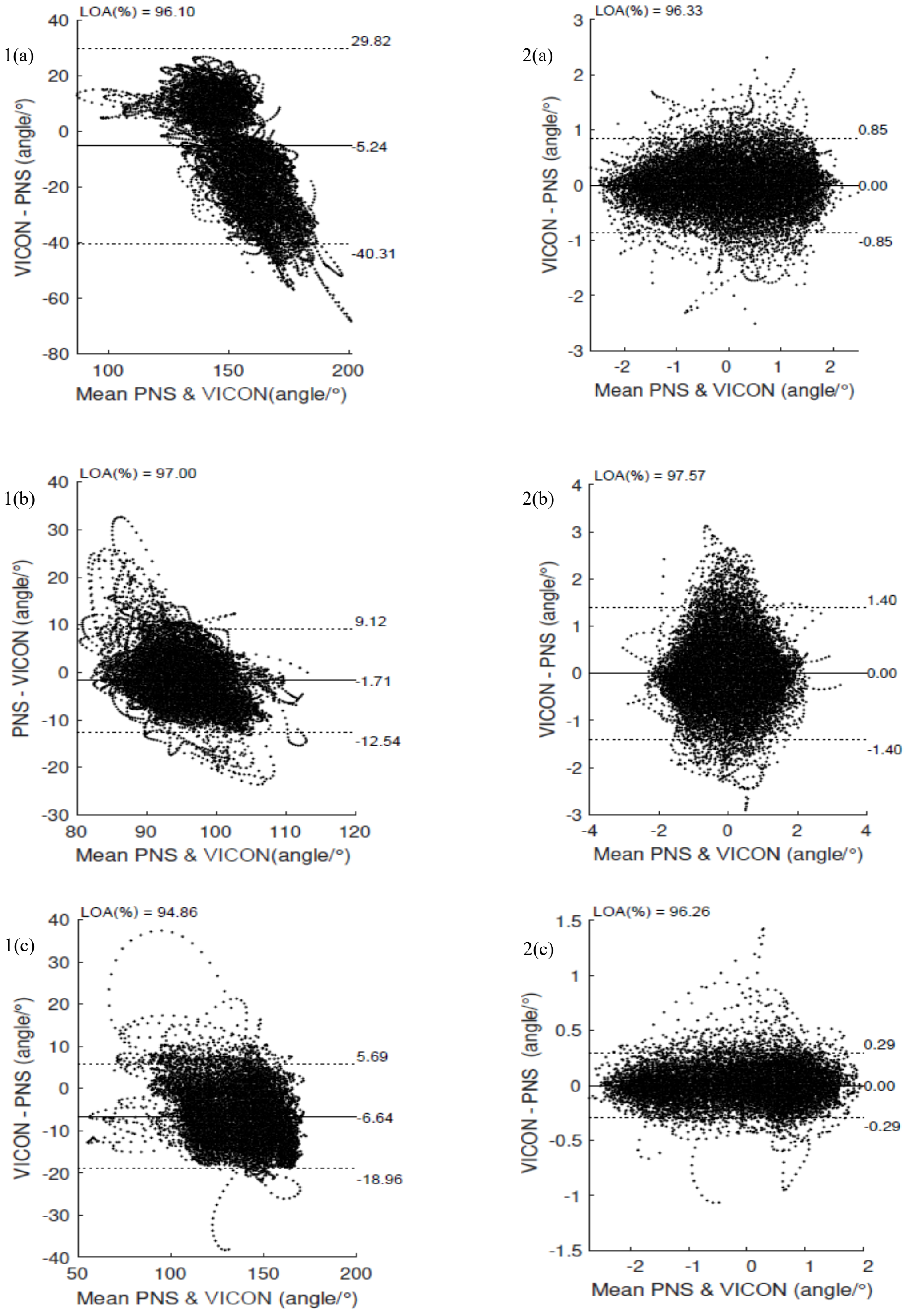

Bland-Altman plots of agreement for (a) hip flexion/extension, (b) hip abduction/adduction and (c) knee flexion/extension for distance jog using (1) raw joint angles and (2) normalized joint angles. Solid horizontal lines represent the mean difference and the dashed horizontal lines represents the 95% limits of agreement (± 1.96 SD).

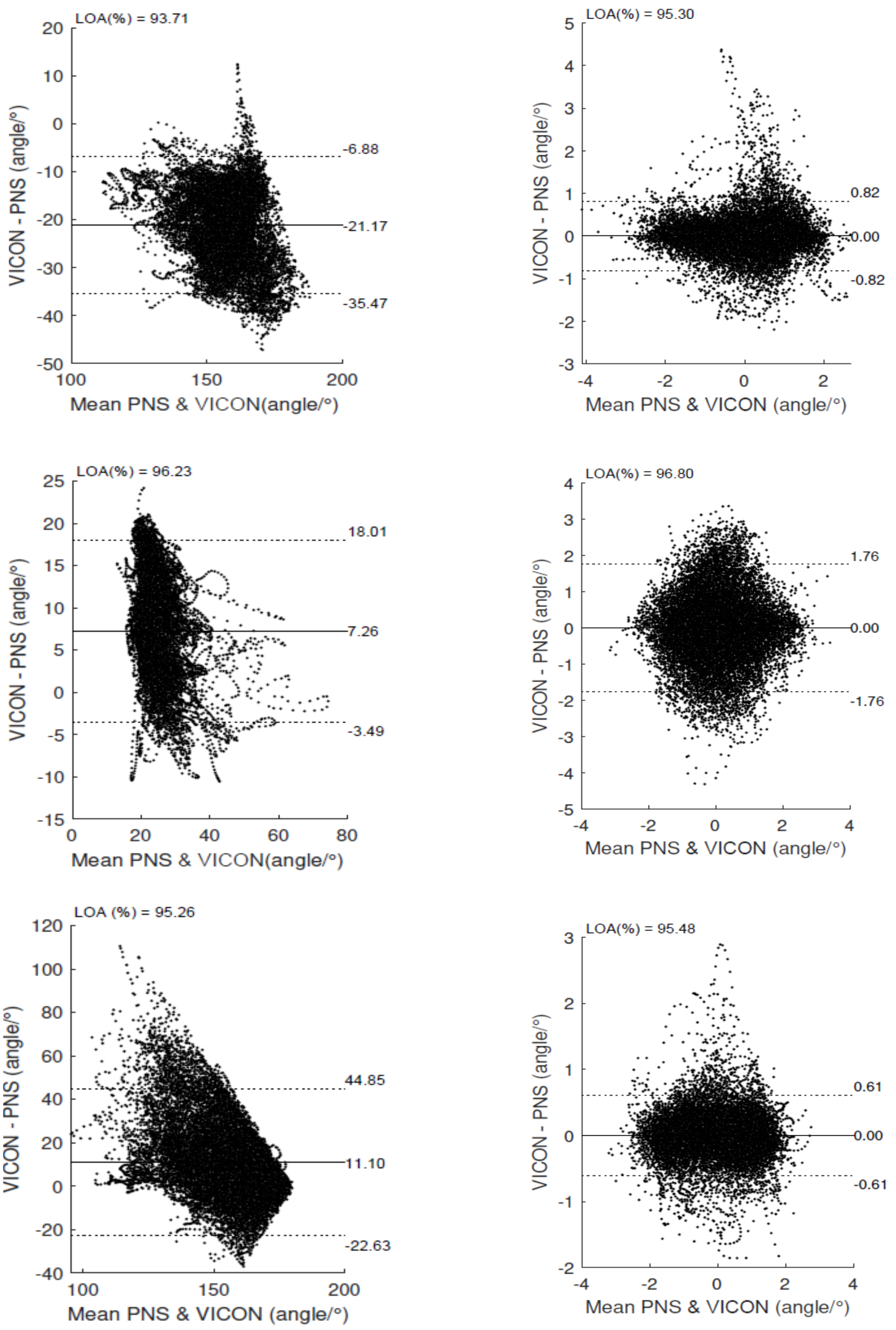

Bland-Altman plots of agreement for (a) elbow flexion/extension, (b) shoulder flexion/extension, (c) shoulder abduction/adduction for distance walk using (1) raw joint angles and (2) normalized joint angles. Solid horizontal lines represent the mean difference and the dashed horizontal lines represents the 95% limits of agreement (± 1.96 SD).

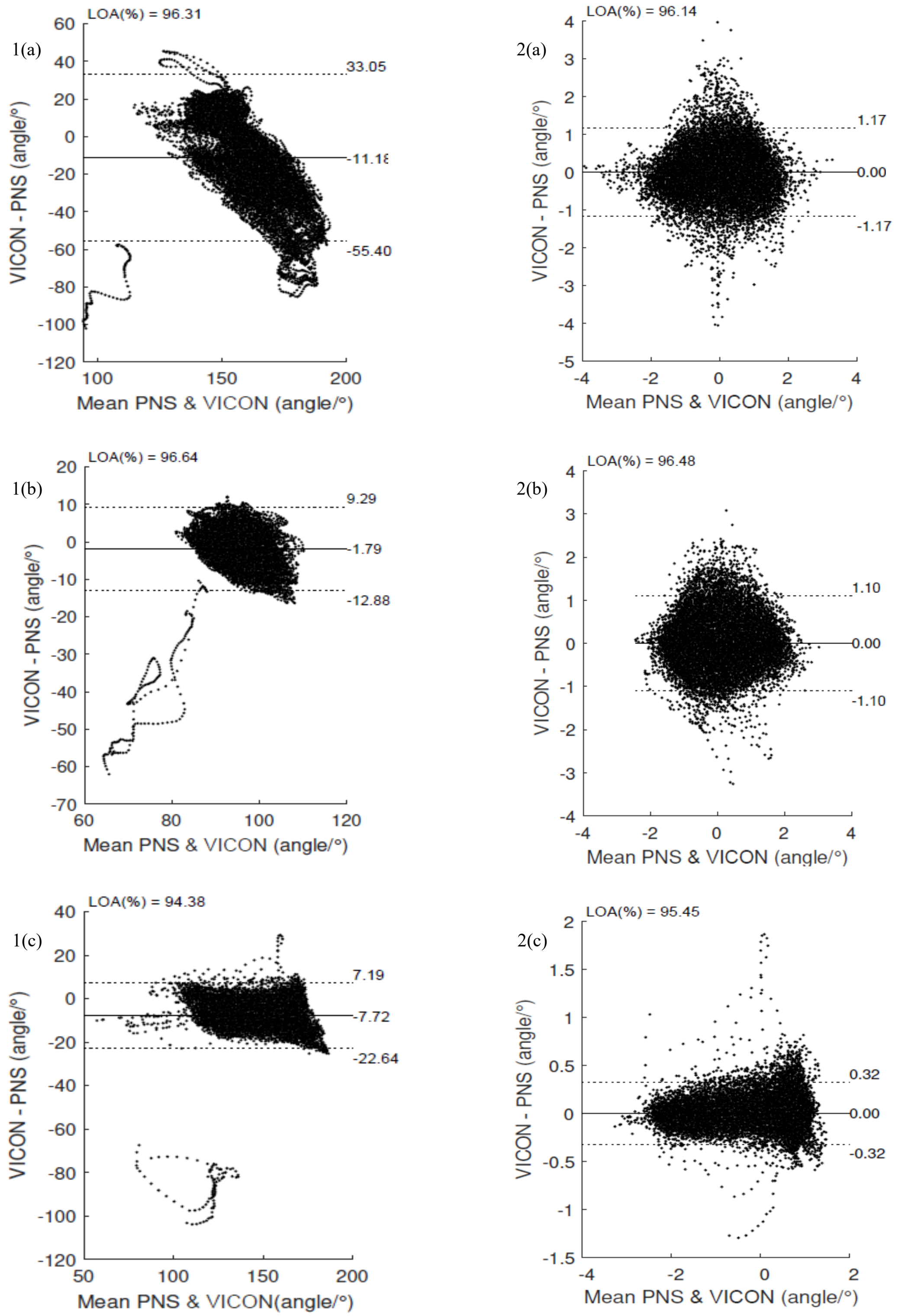

Bland-Altman plots of agreement for (a) hip flexion/extension, (b) hip abduction/adduction and (c) knee flexion/extension for distance walk using (1) raw joint angles and (2) normalized joint angles. Solid horizontal lines represent the mean difference and the dashed horizontal lines represents the 95% limits of agreement (± 1.96 SD).

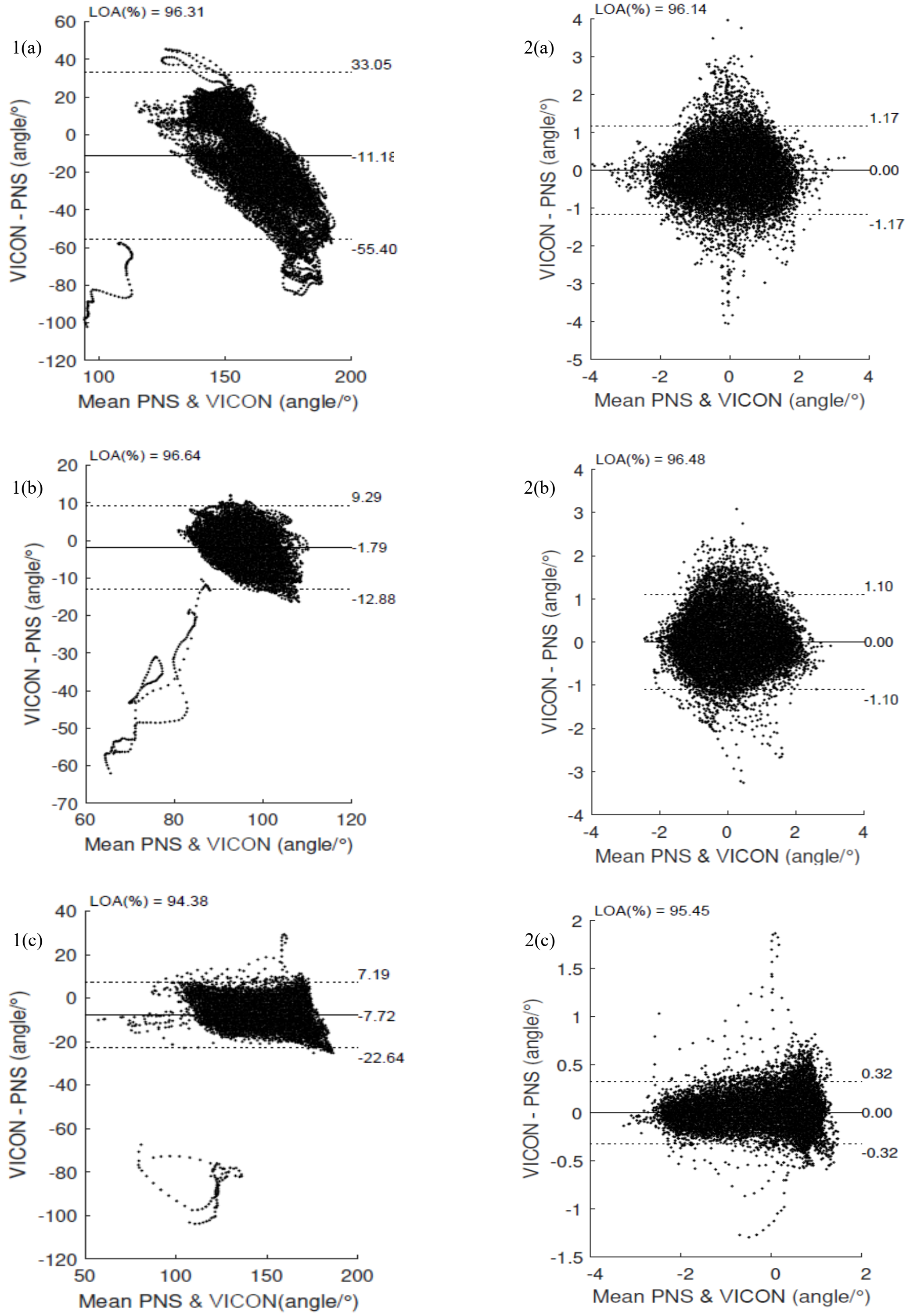

Bland-Altman plots of agreement for (a) elbow flexion/extension, (b) shoulder flexion/extension, (c) shoulder abduction/adduction for distance wrist shot using (1) raw joint angles and (2) normalized joint angles. Solid horizontal lines represent the mean difference and the dashed horizontal lines represents the 95% limits of agreement (± 1.96 SD).

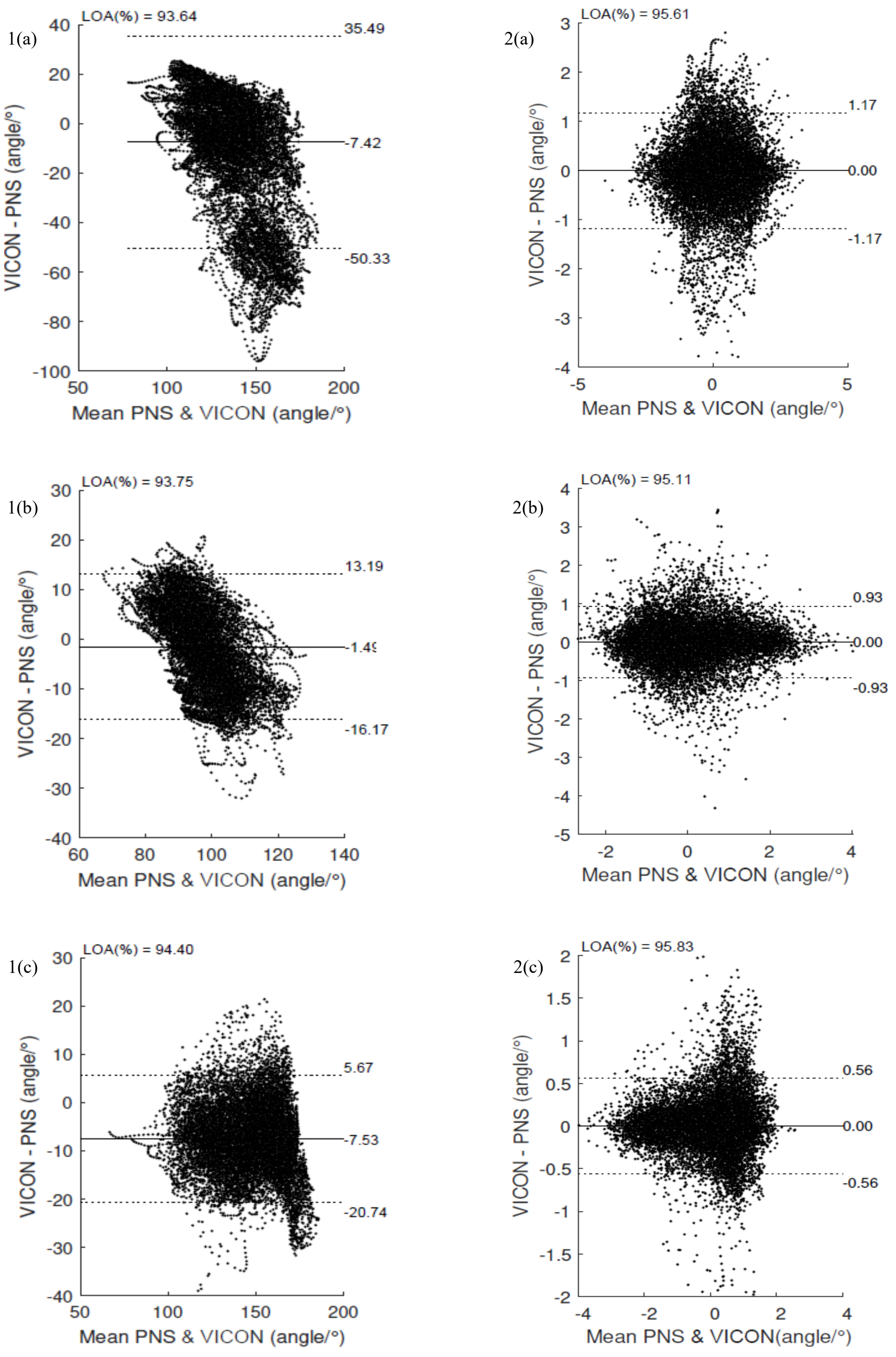

Bland-Altman plots of agreement for (a) hip flexion/extension, (b) hip abduction/adduction and (c) knee flexion/extension for distance wrist shot using (1) raw joint angles and (2) normalized joint angles. Solid horizontal lines represent the mean difference and the dashed horizontal lines represents the 95% limits of agreement (± 1.96 SD).

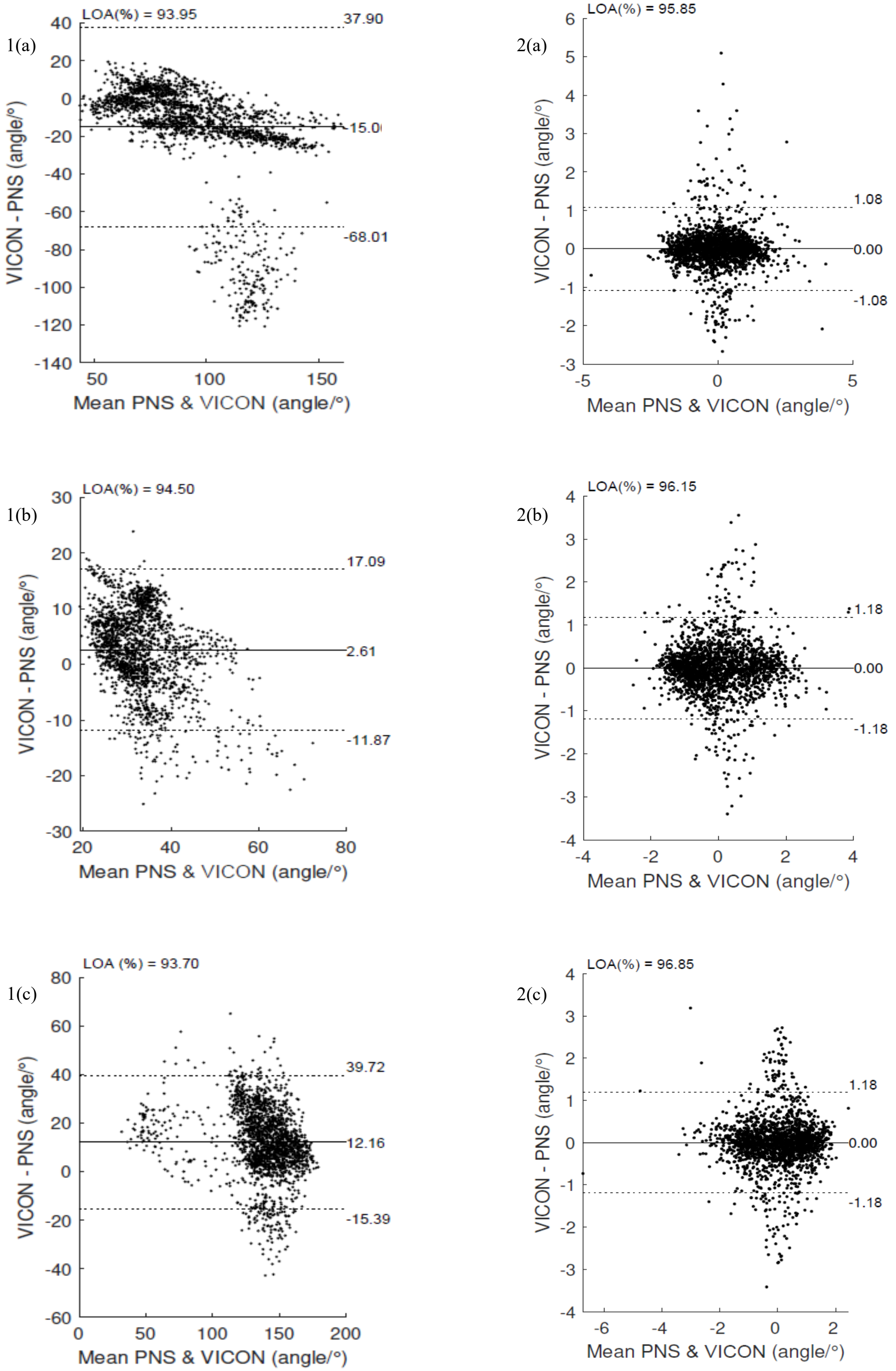

Bland-Altman plots of agreement for (a) elbow flexion/extension, (b) shoulder flexion/extension, (c) shoulder abduction/adduction for stationary jog (1) raw joint angles and (2) normalized joint angles. Solid horizontal lines represent the mean difference and the dashed horizontal lines represents the 95% limits of agreement (± 1.96 SD).

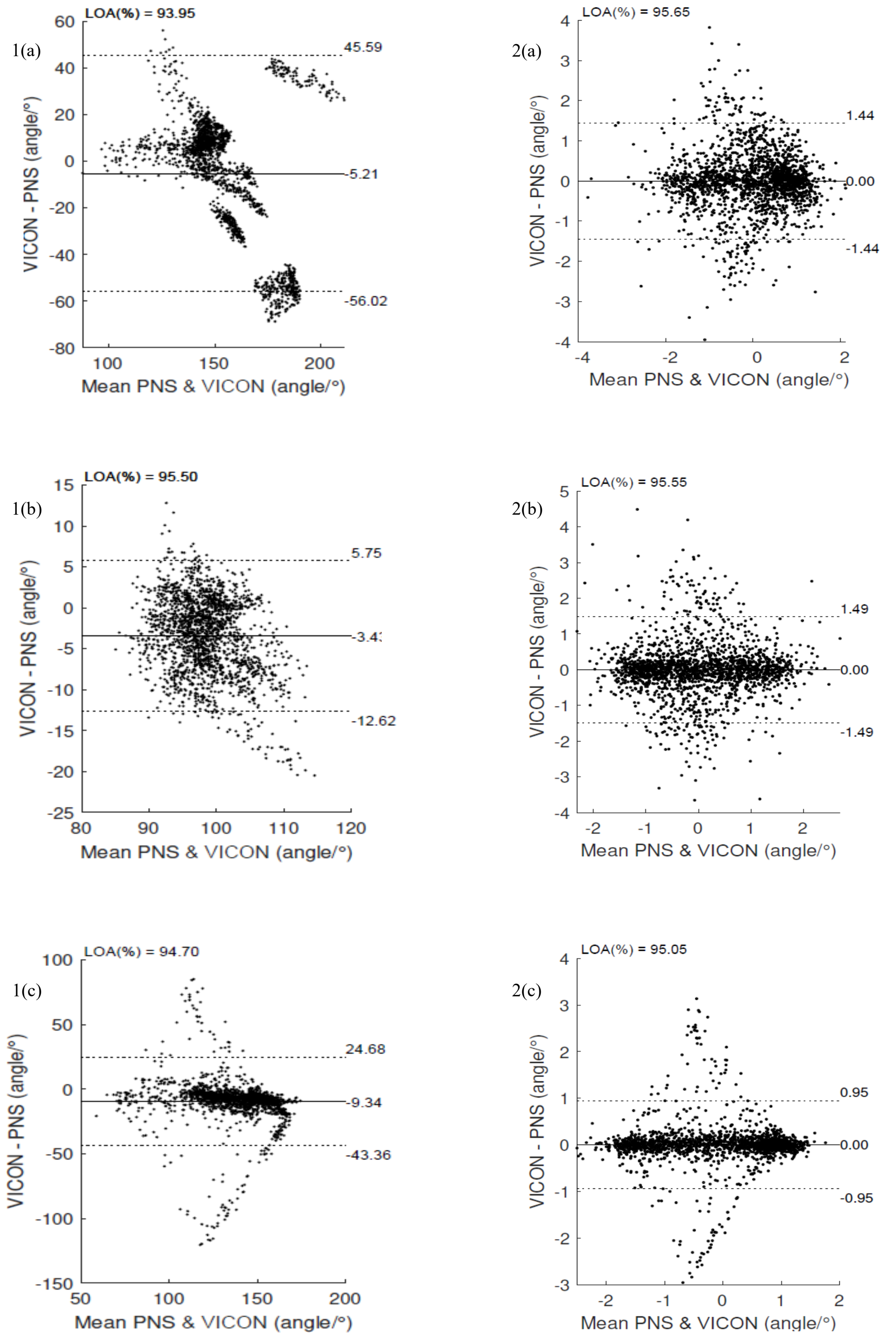

Bland-Altman plots of agreement for (a) hip flexion/extension, (b) hip abduction/adduction and (c) knee flexion/extension for stationary jog (1) raw joint angles and (2) normalized joint angles. Solid horizontal lines represent the mean difference and the dashed horizontal lines represents the 95% limits of agreement (± 1.96 SD).

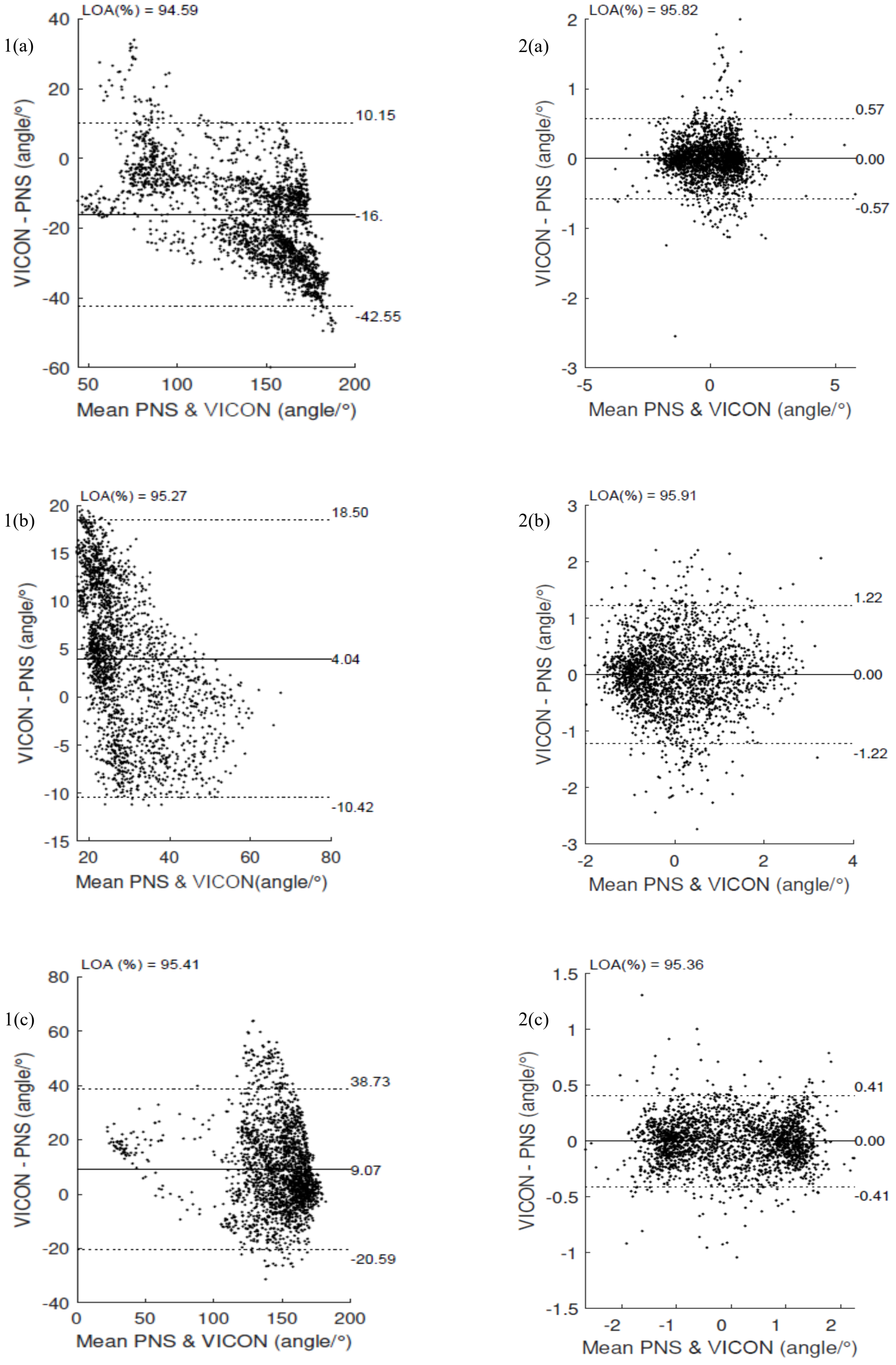

Bland-Altman plots of agreement for (a) elbow flexion/extension, (b) shoulder flexion/extension, (c) shoulder abduction/adduction for stationary walk using (1) raw joint angles and (2) normalized joint angles. Solid horizontal lines represent the mean difference and the dashed horizontal lines represents the 95% limits of agreement (± 1.96 SD).

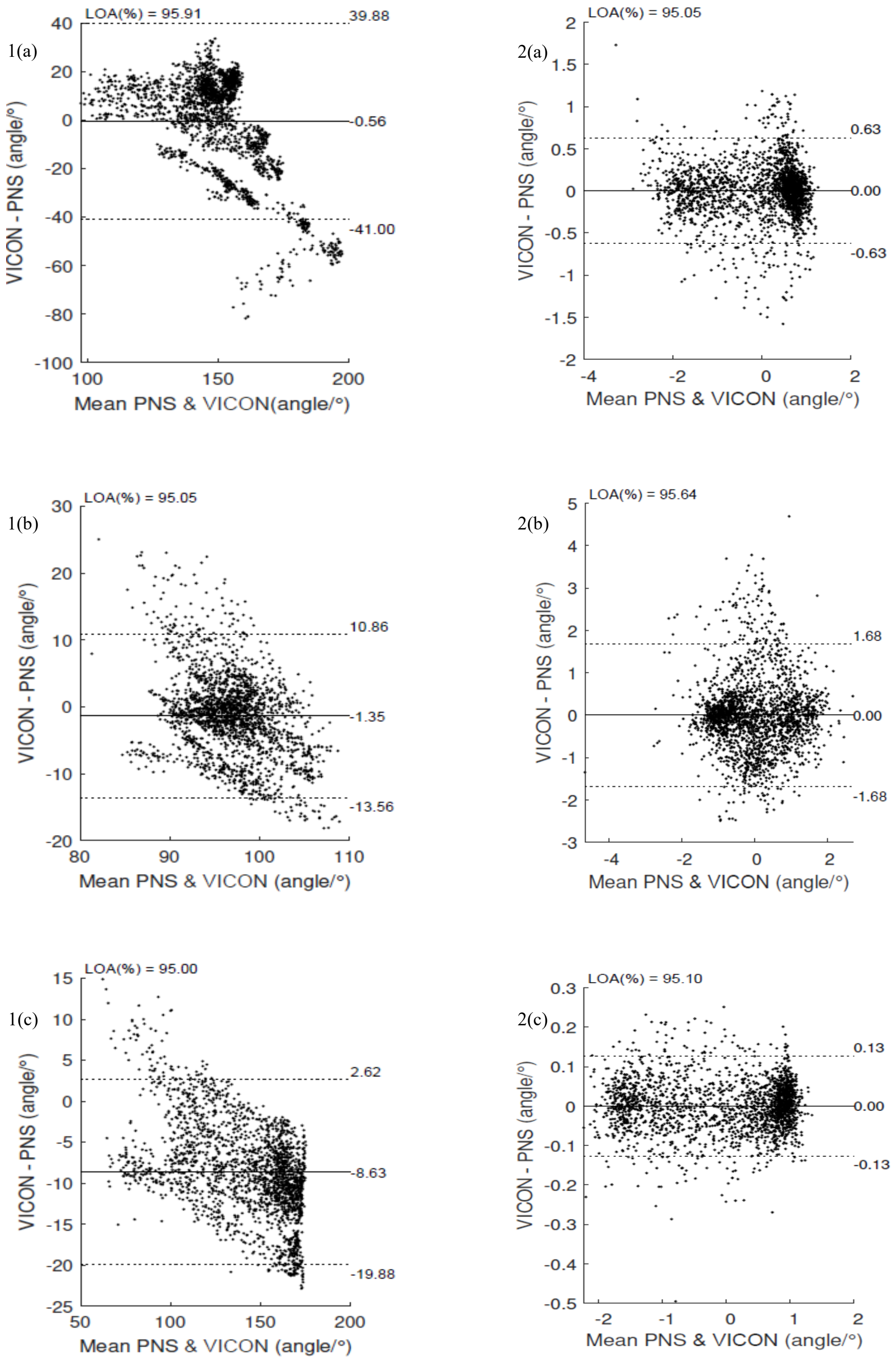

Bland-Altman plots of agreement for (a) hip flexion/extension, (b) hip abduction/adduction and (c) knee flexion/extension for stationary walk using (1) raw joint angles and (2) normalized joint angles. Solid horizontal lines represent the mean difference and the dashed horizontal lines represents the 95% limits of agreement (± 1.96 SD).

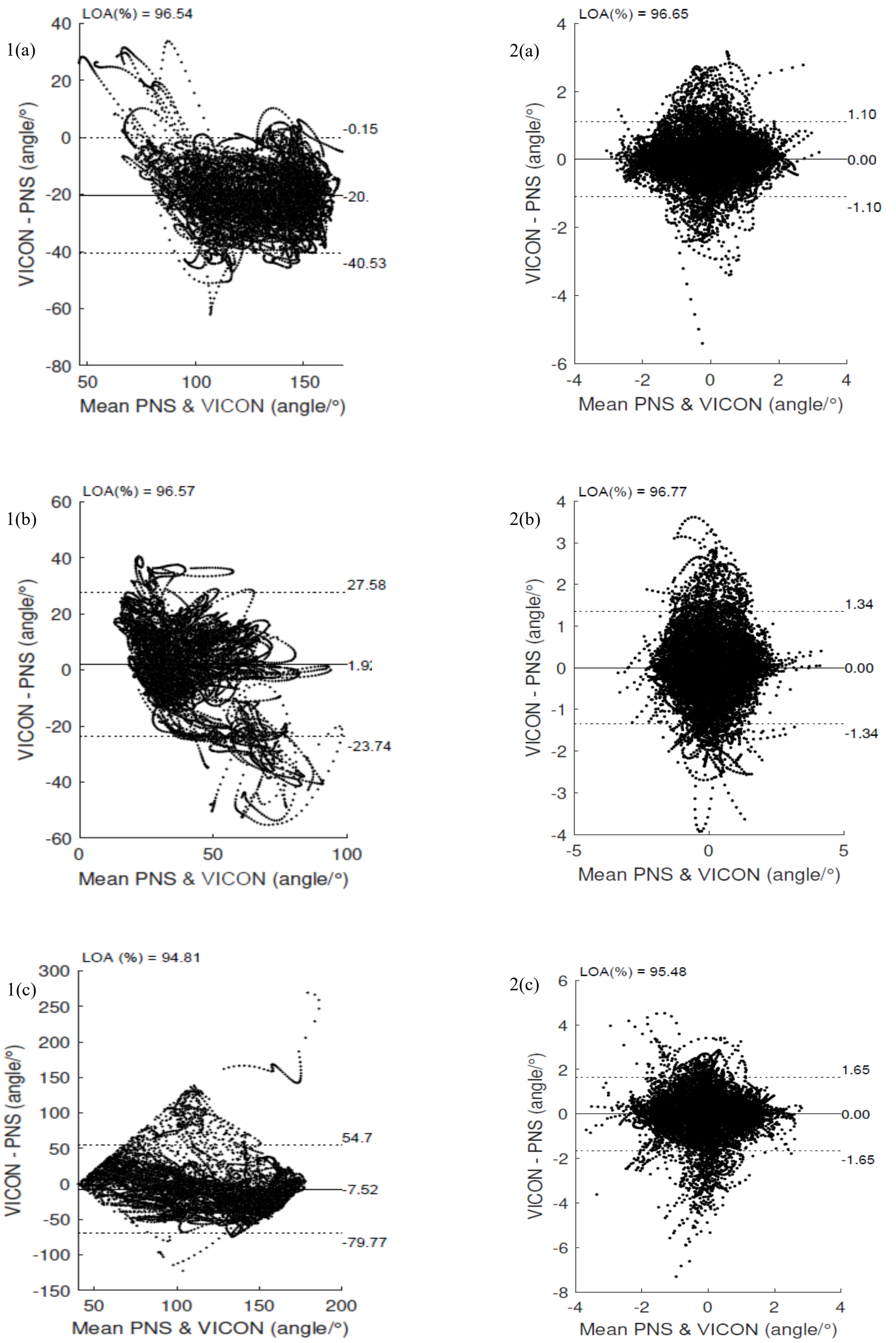

Bland-Altman plots of agreement for (a) elbow flexion/extension, (b) shoulder flexion/extension, (c) shoulder abduction/adduction for stationary wrist shot using (1) raw joint angles and (2) normalized joint angles. Solid horizontal lines represent the mean difference and the dashed horizontal lines represents the 95% limits of agreement (± 1.96 SD).

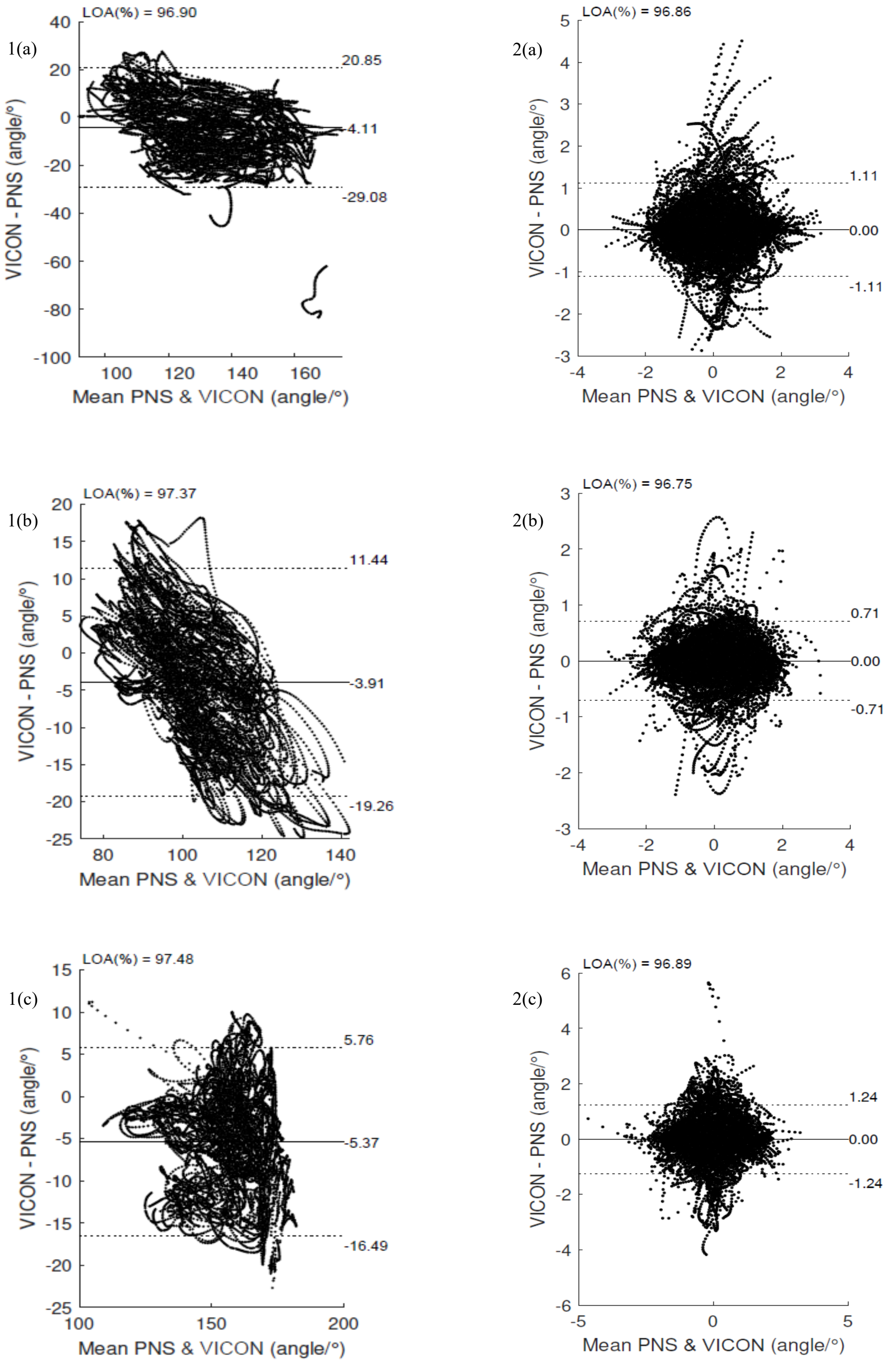

Bland-Altman plots of agreement for (a) hip flexion/extension, (b) hip abduction/adduction and (c) knee flexion/extension for stationary wrist shot using (1) raw joint angles and (2) normalized joint angles. Solid horizontal lines represent the mean difference and the dashed horizontal lines represents the 95% limits of agreement (± 1.96 SD).
