# Supplementary material for: Validation of the Perception Neuron system for full-body motion capture
Source: PLoS One. 2022 Jan 21;17(1):e0262730. doi: 10.1371/journal.pone.0262730 (PMC8782534; doi:10.1371/journal.pone.0262730)
Supplement: Attachment — Submitted filename: PONE-D-20-33330_reviewerComments.pdf [file pone.0262730.s002.pdf]

PLOS ONE

Validation of the Perception Neuron system for full-body motion capture

--Manuscript Draft--

**Manuscript Number:**	PONE-D-20-33330
**Article Type:**	Research Article
**Full Title:**	Validation of the Perception Neuron system for full-body motion capture
**Short Title:**	Validation of the Perception Neuron system for full-body motion capture
**Corresponding Author:**	Corliss Choo<br>Nanyang Technological University National Institute of Education<br>Singapore, SINGAPORE
**Keywords:**	Inertial motion capture; IMUs; Perception Neuron; Vicon; Validation; Full-body motion analysis
**Abstract:**	Recent advancements in Inertial Measurement Units (IMUs) offers the possibility of its use as a cost effective and portable alternative to traditional optoelectronic motion capture systems in analyzing biomechanical performance. One such commercially available IMU is the Perception Neuron motion capture system (PNS). The accuracy of the PNS was tested by Sers, Forrester (1) and was reported to be a valid method for assessing the upper body range of motion to within 5° RMSE. However, testing of the PNS was limited to upper body motion involving functional movement within a single plane. Therefore the purpose of this study is to further validate the Perception Neuron system with reference to a conventional optoelectronic motion capture system (VICON) through the use of dynamic movements (e.g., walking, jogging and a multi-articular sports movement with object manipulation) and to determine its feasibility through full-body kinematic analysis. Validation was evaluated using Pearson's R correlation, RMSE and Bland-Altman estimates. Present findings suggest that the PNS performed well against the VICON motion analysis system with most joint angles reporting a RMSE of < 4° and strong average Pearson's R correlation of 0.85, with the exception of the shoulder abduction/adduction where RMSE was larger and Pearson's R correlation at a moderate level. Bland-Altman analysis revealed that most joint angles across the different movements had a mean bias of less than 10°, except for the shoulder abduction/adduction and elbow flexion/extension measurements. It was concluded that the PNS may not be the best substitute for traditional motion analysis technology if there is a need to replicate raw joint angles. However, there was adequate sensitivity to measure changes in joint angles and would be suitable when normalized joint angles are compared and the focus of analysis is to identify changes in movement patterns.
**Order of Authors:**	Corliss Zhi Yi Choo
	Jia Yi Chow
	John Komar
**Additional Information:**	
**Question**	**Response**
**Financial Disclosure**<br><br>Enter a financial disclosure statement that describes the sources of funding for the work included in this submission. Review the submission guidelines for detailed requirements. View published research articles from *PLOS ONE* for specific examples.<br><br>This statement is required for submission	The author(s) received no specific funding for this work.

Powered by Editorial Manager® and ProduXion Manager® from Aries Systems Corporation

and **will appear in the published article** if the submission is accepted. Please make sure it is accurate.<br><br>**Unfunded studies**<br>Enter: *The author(s) received no specific funding for this work.*<br><br>**Funded studies**<br>Enter a statement with the following details:<br>• Initials of the authors who received each award<br>• Grant numbers awarded to each author<br>• The full name of each funder<br>• URL of each funder website<br>• Did the sponsors or funders play any role in the study design, data collection and analysis, decision to publish, or preparation of the manuscript?<br>• **NO** - Include this sentence at the end of your statement: *The funders had no role in study design, data collection and analysis, decision to publish, or preparation of the manuscript.*<br>• **YES** - Specify the role(s) played.<br><br>* typeset	
**Competing Interests**<br><br>Use the instructions below to enter a competing interest statement for this submission. On behalf of all authors, disclose any competing interests that could be perceived to bias this work—acknowledging all financial support and any other relevant financial or non-financial competing interests.<br><br>This statement **will appear in the published article** if the submission is accepted. Please make sure it is accurate. View published research articles from *PLOS ONE* for specific examples.	The authors have declared that no competing interests exist.

**NO authors have competing interests**<br><br>Enter: *The authors have declared that no competing interests exist.*<br><br>**Authors with competing interests**<br><br>Enter competing interest details beginning with this statement:<br><br>*I have read the journal's policy and the authors of this manuscript have the following competing interests: [insert competing interests here]*<br><br>* typeset	
**Ethics Statement**<br><br>Enter an ethics statement for this submission. This statement is required if the study involved:<br><br>• Human participants<br>• Human specimens or tissue<br>• Vertebrate animals or cephalopods<br>• Vertebrate embryos or tissues<br>• Field research<br><br>Write "N/A" if the submission does not require an ethics statement.<br><br>General guidance is provided below. Consult the submission guidelines for detailed instructions. **Make sure that all information entered here is included in the Methods section of the manuscript.**	Human Subject Research<br>NTU Institutional Review Board<br>IRB-2019-03-005<br>Written consent was obtained

Powered by Editorial Manager® and ProduXion Manager® from Aries Systems Corporation

**Format for specific study types**<br><br>**Human Subject Research (involving human participants and/or tissue)**<br>• Give the name of the institutional review board or ethics committee that approved the study<br>• Include the approval number and/or a statement indicating approval of this research<br>• Indicate the form of consent obtained (written/oral) or the reason that consent was not obtained (e.g. the data were analyzed anonymously)<br><br>**Animal Research (involving vertebrate animals, embryos or tissues)**<br>• Provide the name of the Institutional Animal Care and Use Committee (IACUC) or other relevant ethics board that reviewed the study protocol, and indicate whether they approved this research or granted a formal waiver of ethical approval<br>• Include an approval number if one was obtained<br>• If the study involved *non-human primates*, add *additional details* about animal welfare and steps taken to ameliorate suffering<br>• If anesthesia, euthanasia, or any kind of animal sacrifice is part of the study, include briefly which substances and/or methods were applied<br><br>**Field Research**<br><br>Include the following details if this study involves the collection of plant, animal, or other materials from a natural setting:<br>• Field permit number<br>• Name of the institution or relevant body that granted permission	
**Data Availability**<br><br>Authors are required to make all data underlying the findings described fully available, without restriction, and from the time of publication. PLOS allows rare exceptions to address legal and ethical concerns. See the PLOS Data Policy and FAQ for detailed information.	Yes - all data are fully available without restriction

A Data Availability Statement describing where the data can be found is required at submission. Your answers to this question constitute the Data Availability Statement and **will be published in the article**, if accepted.<br><br>**Important:** Stating 'data available on request from the author' is not sufficient. If your data are only available upon request, select 'No' for the first question and explain your exceptional situation in the text box.<br><br>Do the authors confirm that all data underlying the findings described in their manuscript are fully available without restriction?	
**Describe where the data may be found in full sentences. If you are copying our sample text, replace any instances of XXX with the appropriate details.**<br><br>• If the data are **held or will be held in a public repository**, include URLs, accession numbers or DOIs. If this information will only be available after acceptance, indicate this by ticking the box below. For example: *All XXX files are available from the XXX database (accession number(s) XXX, XXX.*).<br>• If the data are all contained **within the manuscript and/or Supporting Information files**, enter the following: *All relevant data are within the manuscript and its Supporting Information files.*<br>• If neither of these applies but you are able to provide **details of access elsewhere**, with or without limitations, please do so. For example:<br><br>*Data cannot be shared publicly because of [XXX]. Data are available from the XXX Institutional Data Access / Ethics Committee (contact via XXX) for researchers who meet the criteria for access to confidential data.*<br><br>*The data underlying the results presented in the study are available from (include the name of the third party*	All relevant data are within the manuscript and its Supporting Information files.<br><br>Any other data associated with the manuscript will be held on the NIE data repository.

Powered by Editorial Manager® and ProduXion Manager® from Aries Systems Corporation

and contact information or URL).<br>• This text is appropriate if the data are owned by a third party and authors do not have permission to share the data.<br><br>* typeset	
Additional data availability information:	Tick here if the URLs/accession numbers/DOIs will be available only after acceptance of the manuscript for publication so that we can ensure their inclusion before publication.; Tick here if your circumstances are not covered by the questions above and you need the journal’s help to make your data available.

Powered by Editorial Manager® and ProduXion Manager® from Aries Systems Corporation

Manuscript Click here to access/download;Manuscript;Validation of the PN for full-body motion capture.docx

Validation of the Perception Neuron system for full-body motion capture

Corliss Zhi Yi Choo[1], Jia Yi Chow[1], John Komar[1]

[1] Physical Education and Sports Science, National Institute of Education, Nanyang Technological University, Singapore, Singapore

* corliss.choo@nie.edu.sg

Abstract

Recent advancements in Inertial Measurement Units (IMUs) offers the possibility of its use as a cost effective and portable alternative to traditional optoelectronic motion capture systems in analyzing biomechanical performance. One such commercially available IMU is the Perception Neuron motion capture system (PNS). The accuracy of the PNS was tested by Sers, Forrester (1) and was reported to be a valid method for assessing the upper body range of motion to within 5° RMSE. However, testing of the PNS was limited to upper body motion involving functional movement within a single plane. Therefore the purpose of this study is to further validate the Perception Neuron system with reference to a conventional optoelectronic motion capture system (VICON) through the use of dynamic movements (e.g., walking, jogging and a multi-articular sports movement with object manipulation) and to determine its feasibility through full-body kinematic analysis. Validation was evaluated using Pearson's R correlation, RMSE and Bland-Altman estimates. Present findings suggest that the PNS performed well against the VICON motion analysis system with most joint angles reporting a RMSE of < 4° and strong average Pearson's R correlation of 0.85, with the exception of the shoulder abduction/adduction where RMSE was larger and Pearson's R correlation at a moderate level. Bland-Altman analysis revealed that most joint angles across the different movements had a mean bias of less than 10°, except for the shoulder abduction/adduction and elbow flexion/extension measurements. It was concluded that the PNS may not be the best substitute for traditional motion analysis technology if there is a need to replicate raw joint angles. However, there was adequate sensitivity to measure changes in joint angles and would be suitable when normalized joint angles are compared and the focus of analysis is to identify changes in movement patterns.

Introduction

Traditionally, motion capture has been collected through the use of high-speed optical systems which allows for dynamic movements to be captured and analysed (2, 3). These systems serve as a reliable and suitable method to measure complex movements and are often considered as the gold standard in motion capture, providing an estimated accuracy (RMS error) of up to < 1.00° and <1.50° for static and dynamic measurements respectively (3-5). An example of a high-speed optical system is the VICON motion analysis system (Oxford Metrics Group Ltd., Oxford, UK). For such systems, passive reflective markers are placed on specific body landmarks which reflect light back into the sensors of the cameras and the system computes the 3D positions of the makers within the environment using the triangulation method (2, 3, 5).

3D optoelectronic motion capture systems have improved significantly over the years due to higher camera resolution, better calibration techniques and improvements in manufacturer tracking software which allows for more accurate marker tracking and greater post processing capabilities (2, 6). With these changes in technology, consistency in accuracy by such systems have showed a nearly three-fold improvements in agreement over the last 20 years with no systems exceeding an error of 1.0mm (6). Due to its validity and reliability as compared to other motion capture systems (3), optoelectronic motion capture systems have been extensively used in a variety of field, ranging from functional movement tasks (7, 8) to sports performance (9, 10) and injury prevention (11, 12), thus contributing meaningful information and advancements to the field of biomechanics over the years (2).

Although optical based systems have been commonly used for motion analysis, they are still expensive, have somewhat limited portability and typically require movement to be recorded within the confines of a laboratory environment (1, 5, 13). Set-up and calibration also involves a lengthy preparation process (14). Furthermore during data collection, markers have to be in line-of-sight of the

cameras as any occlusion or alterations to camera positions will affect the reliability of the data (3). Thus even though optical based systems are considered as the gold standard with consistently accurate measurements, the desire by researchers to collect accurate and reliable kinematic in applied situations for research and for the routine analysis of movements within normal training environments without being obstructed by environmental restrictions and lengthy processing time has increasingly driven researchers to search for alternatives (2, 15).

although true, most systems have enough cameras that this is not a problem

For most lab collections obstruction is not an issue. Is there a specific application you have in mind where it is?

One such alternative is the inertial measurement unit (IMU). IMUs are devices that comprise of a gyroscope, an accelerometer and magnetometer and measures the motion of the user through embedded data fusion, human body dynamics and physical engine algorithms which allows for the estimation of kinematic data of body segments within a 3D space to be obtained (4, 16). These IMUs allow users to capture and record real-time motion with a fast set-up process and minimal space restrictions thus increasing the portability and allowing movement capture to be conducted within real-life environments (16). Hence, IMUs could potentially serve as a solution to the limitations that optical based motion capture systems face.

Importantly, recent advancements in IMUs offers the possibility of its use as a cost effective, portable alternative to analyzing biomechanical performance in an efficient way (4, 17, 18). In general a combination of higher sampling rate, better sensor fusion algorithms and lower drift have resulted in increased accuracy of the data collected by these sensors (19). As such IMUs have been used in various applications in sports, such as technique identification and performance, tracking athletes' performance and for injury screening (3, 14, 20). One example of technique identification and performance is a study conducted by Bosch, Shoaib (21), where researchers were able to distinguish between experienced and novice rowers by simply placing sensors on the lower leg, the lower back and upper back and analyzing consistency in postural angles and timing of the strokes. With respect to tracking athletes' performance, Shepherd, Giblin (22) used IMUs effectively to monitor shooting kinematics and coordination patterns of

Is this compared to earlier IMUs? What's the accuracy? You cited the accuracy of the optical mocap systems earlier.

netball athletes. This provided coaches and athletes with actionable insights to develop practices to achieve more consistent forearm angle at ball release and increase their chances of scoring during a game. IMUs have also been used for injury screening and prevention by evaluating movements and specifically to identify the presence of abnormal movements during performance (23). They allow scientists to quantitatively monitor and detect movement kinematics or volumes of training which has been linked to overuse injuries and hence allow them to issue preventive warnings (23-25). As seen from the studies above, information obtained from these IMUs could be utilized by sports scientists and professional coaches in a myriad of ways and within ecological contexts (17, 26, 27). Furthermore with a combination of lower costs, greater ease of use, increased portability for use in the field, it could eventually drive the use of these IMUs in sports not only exclusively for elite athletes but for a much wider target audience (14, 28). However, accuracy of the IMUs depends on the specific sensors used, the software algorithms of the tested IMU, and the measured joint and task that is being performed (19). Therefore it is necessary that specific validation of each new IMU system is completed before any IMU can be used routinely in assessments (19).

One such commercially available IMU is the Perception Neuron motion capture system (PNS) (Perception Neuron, Noitom, Miami, FL, USA). It is an adaptable and affordable motion capture system offering user-friendly technology that was developed to analyse movements for various industries from film makers to game developers, biomechanics researchers and sports and medical analysts. Some of the benefits of the PNS includes its ease of use since the sensors are interchangeable and also the portability of the system due to the ability to rely on a portable battery for its energy needs. In addition, the PNS has the capacity to record the data locally on a memory card or transfer the data through WiFi instead of a wired connection.

Currently, validation of the PNS is still limited. One such validation study was conducted by Sers, Forrester (1), where the accuracy of joint angles measurements acquired by the PNS was referenced

against a gold standard optoelectronic systems (VICON). Movements analysed included neck flexion/extension, neck lateral flexion, neck rotation, torso flexion/extension, torso later flexion, torso rotation and shoulder abduction. Participants were tasked to perform each movement twice and at self-selected fast and slow speeds. Results suggests that the PNS is a valid method to assess the majority of the upper body range of motion during static movements with systematic and random biasness for majority of the range of motion differences being below 4.5°, with an exception for the knee extension where values were 6.1 - 9.0°. However Sers, Forrester (1) only analysed the validity of the PNS using only postural angular kinematics of the upper body.

uncommon word bias is probably preferable

Therefore, the purpose of the study was to further validate the PNS with reference to a conventional optoelectronic motion capture system for full-body human movement during more dynamic movements and to determine its feasibility in a comprehensive full body kinematic analysis. Specifically, performance of the PNS was assessed using kinematic measures (e.g., joint angles) in comparison to the VICON motion analysis system using a series of full-body human movements. Walking, jogging and floorball wrist shots were used as a proposed task vehicle as these activities are predicated on its feature as a discrete multi-articular tasks that involves the coordination of multiple body parts, joints and limb segments with object manipulation. The wrist shot was specifically chosen out of the many shots available within floorball as it is the most common shot used by players to score a goal and it is executed at a fast pace. The inclusion of these movements allows the testing of the PN systems' ability to measure kinematic data during dynamic and fast paced tasks, which was previously not examined in the study undertaken by Sers, Forrester (1). It is hypothesized that measurements from the PNS would be comparable to the measurements from a traditional optoelectronic motion capture analysis system (VICON), thus validating the use of the PNS as an alternative for full-body human motion capture. By establishing the validity of the PNS, it ensures that the system can adequately measure full-body movement. The PNS could then potentially be used as an alternative to the current optical motion

Be consistent - use either PN system(s) or PNS throughout the paper

systems during sports biomechanical applications. This could help mitigate the restrictions of the traditional 3D optical motion system which are expensive and better allow collection of data within real-world settings outside the restricted confines of a laboratory.

Methods

Participants

Ten (spell out number if it starts the sentence or else rearrange)

10 participants (7 males and 3 females) with a mean age of 26 ± 2 years, mean height of 1.70 ± 0.09 m and mean weight of 60.48 ± 11.91 kg participated in this study. Written participant consent was obtained prior to the start of any data collection. Human ethics approval was granted by the University Institutional Review Board. Exclusion criterion was musculoskeletal injury, illnesses, or diseases for the past 6 months prior to the time of testing, mainly to ensure safety for the participants.

Instrumentation

Movement data was collected simultaneously with the PNS and an eight-camera VICON MX40 optical motion capture system recording at 120Hz each. Participants' movement were also captured and recorded via two digital video cameras (CASIO EXILIM EX100, Tokyo, Japan) from two planes of observation (frontal and sagittal). The VICON optoelectronic motion analysis system was chosen as it is

I'm not sure this is objectively clear. Perhaps you could say is "a widely used system"

the and is considered as the current laboratory gold-standard with a high accuracy during dynamic trials (1, 19, 29). Was this the result in Topley & Richards (2020)?

Spell out at start of sentence

reflective markers (19-mm) were placed on anatomical landmarks based on the Full-body modeling with Plug-in Gait (Vicon Peak, Oxford, UK). The PNS consisted of 17 IMUs placed at specific landmarks in accordance to PNS guidelines as shown in Fig 1. The PNS suit was connected to a computer

Does the suit increase or decrease the soft tissue artifact from the markers? Is this a source of uncertainty?

Does the USB connect affect peoples' movement as old EMG system wires used to?

through a USB connection and the Axis Neuron software (AXIS Neuron, Noitom, Miami, Florida, USA) was used to view the data in real-time.

Fig 1. Perception Neuron IMU sensor configuration.

Calibration of both systems was also completed prior to each movement recording. The PNS was calibrated according to the manufacturer's instructions using a four-step calibration process (see Table 1) and was zeroed after five repetitions of each task to remove any errors. The VICON motion analysis system was calibrated using the T-pose.

Table 1. Perception Neuron 4-step calibration.

**Posture calibration**	**Details**
1. Steady pose	Participant to be seated and to remain as still as possible
2. A-pose	Participant to place palms down on the side of the thighs and keep feet parallel
3. T-pose	Participants to have shoulders abducted by 90º with the palms facing the floor
4. S-pose	Participants to bend knees approximately 45º and place arms in front and position them parallel to the floor

Protocol

All participants underwent one test session. Anthropometric measurements including height, weight, leg length, knee width, ankle width, elbow width and hand thickness was recorded from every participant at the start of the session. Participants were then suited up in the PN 17-neuron full body suit and the 39 19-mm retroreflective markers for the VICON system were placed on anatomical landmarks according to the Plug-in-Gait full-body model (VICON Motion Systems, Oxford Metrics Group Ltd) and held in place using medical grade double-sided adhesive tape as seen in Fig 2.

Fig 2. Participant set-up with both PNS and VICON markers front and back. The red squares indicates the PN sensor positions.

Participants were required to perform a series of tasks. Description of these can be found in Table 2. Participants performed 10 repetitions for each task. During the performance of the tasks, care was taken to ensure that participants' movement were not obstructed in any way as a result of the reflective markers and the PNS.

Table 2. Description of tasks to be performed.

**Task**	**Description**
Stationary walk	Participants to swing their arms, bring their knees up and feet back down to the same point where they took off from at a self-determined walking pace to mimic the walking movement. Participants performed the task whilst remaining within a 90 x 90 cm demarcated spot on the lab floor
Distance walk	Participants to walk along a 3m line on the lab floor at a self-determined walking pace
Stationary jog	Participants to swing their arms, bring their knees up and feet back down to the same point where they took off from at a self-determined jogging pace to mimic the jogging movement. Participants performed the task whilst remaining within a 90 x 90 cm demarcated spot on the lab floor
Distance jog	Participants to walk along a 3m line on the lab floor at a self-determined jogging pace
Stationary floorball wrist shot	Participants to stand 4m in front of an open goal and take shots towards the goal

Moving wrist shot	Participants to stand 7m in front of an open goal, dribble the ball along a 3m line and take a shot towards goal once they are 4m in front of the goal

Data analysis

Data collected from the PNS were exported as a processed bvh file. Displacement of the VICON markers in 3D space were reconstructed and manually labelled for all trials using VICON Nexus 2.9.0. Joint angle data from both systems were calculated from the 3D coordinates using a custom script programmed using MATLAB (R2018b, Mathworks Inc., Natrick, MA USA) which computes both data on the same basis in order to ensure that data from both systems are comparable. Elbow flexion/extension, shoulder flexion/extension, shoulder abduction/adduction, hip flexion/extension, hip abduction/adduction and knee flexion/extension angles were calculated from both systems and comparisons between measurements obtained from the two systems were made. These flexion angles were adapted based on the study of Lee, Chow (30) and Lazzaeri, Kayser (9). Subsequently, joint angles from both devices were smoothed using the “sgolayfilt” function with a polynomial function of degree 3 in MATLAB.

Natick

To compare the joint angles from the VICON and PNS, Pearson’s correlation and RMSE were evaluated for all movements on the raw joint angles. Pearson correlation was used to test the linear relationship between the joint angle measurement from PNS and VICON system. Correlation values were interpreted as such (in either direction positive or negative direction): 0 - 0.3 represents a weak relationship, 0.3 - 0.7 represents a moderate relationship and 0.7 - 1.0 represents a strong relationship. All statistical analyses were completed using MATLAB.

Reference? Is this Cohen?

RMSE was used to compare the two methods as a measure of fit (31),

$$RMSE = \sqrt{\frac{\sum(X-Y)^2}{n}}$$

X(t) Y(t) Indicate that these are time dependent and that you are summing over time.

Where X is the value from the PN suit, Y is the observed value from VICON and n is the total number of samples.

Group averages and SD were presented for Pearson correlation and RMSE.

The construction of Bland-Altman plots (32, 33) was another way to determine the relationship between the two measurement systems. Correlation exploration between the error and the mean value was included to examine for the existence of heteroscedasticity (34). Mean bias and limits of agreement (LOA) were calculated according to Bland and Altman (33) for comparisons on raw joint angles to check for systematic errors. LOA was calculated as such,

$$LOA = \bar{d} \pm 1.96SD$$

where $\bar{d}$ refers to the mean difference and SD refers to the standard deviation.

LOA (%) was also used to check for the agreement between joint angle calculations from the two measurement systems. LOA (%) was defined as the percentage of readings that lie within the limits of agreement. This was calculated for both raw and normalized joint angles.

Results

Table 3 contains the Pearson's R correlation between the PNS and VICON joint angle calculations. All correlation values were statistically significant. Most values showed strong positive correlation with the exception of hip abduction/adduction during stationary walk at 0.63 ± 0.40, shoulder flexion/extension during distance walk with a value of 0.59 ± 0.33 and shoulder abduction/adduction during stationary wrist shot and during distance walk with a correlation of 0.58 ± 0.50 and 0.66 ± 0.43 respectively. Correlation was strong for knee flexion/extension across all movements with correlation ranging between 0.80 and 0.99. Distance wrist shot shoulder also not a strong correlation.

Table 4 displays the RMSE comparisons between the PNS and VICON joint angle calculations on the raw values. RMSE values for raw values were generally below 4° for all joint angles and across all movements except for shoulder abduction/adduction for all movements.

Table 5a and 5b includes the mean bias and limits of agreement (LOA) for each joint angle and across all movements. The elbow flexion/extension showed largest mean bias of 20.62° during distance wrist shots. Whereas the hip flexion/extension had the least differences as compared to the other joint angles with a mean bias of 0.56° during stationary walk. There was a trend for joint angles to be overestimated using the PNS as compared to the VICON across movements. Only the angles for shoulder flexion/extension and shoulder abduction/adduction was underestimated. Existence of heteroscedasticity was found for most joint angles and across all movements. Bland-Altman plots for all joint angles and all movements can be found in Fig 3. This is only for flexion/extension for the wrist shot.

Table 6 shows the LOA (%) between the PNS and VICON joint angle calculations for both raw and normalized values. LOA (%) for raw values ranged from 92.61% to 97.48% across all movements while LOA (%) for normalized values was higher with almost all being above 95% with the exception of shoulder abduction/adduction during distance wrist shots at 94.47%. Fig 3 shows the comparison

between Bland-Altman plots using raw and normalized joint angles. In addition, it was observed that joint angle wave characteristics were more similar when using normalized rather than the raw joint angles. An example of this is depicted in Fig 4(a) and 4(b).

How were data normalized?

Fig 3. Bland-Altman plot of agreement for hip flexion/extension for distance wrist shot using (a) raw joint angles (b) normalized joint angles for all participants. Solid horizontal lines represent the mean difference and the dashed horizontal lines represents the 95% limits of agreement (± 1.96 SD).

Fig 4. Hip flexion/extension angles during a stationary wrist shot using raw joint angle values (a) and Hip flexion/extension angles during a stationary wrist shot using normalized joint angle values (b). Data shown is from one trial from one participant.

Table 7 includes the range of movement tested for all joint angles across the different movements.

Table 3. Pearson's correlation comparison between PNS and VICON.

Joint Angle	Stationary walk	Distance walk	Stationary jog	Distance jog	Stationary wrist shot	Distance wrist shot
Elbow (flex/ext)	0.96	0.91	0.85	0.94	0.84	0.85
SD	0.06	0.17	0.31	0.08	0.25	0.17
p-value	< 0.001	0.0023	0.026	< 0.001	0.014	< 0.001
Shoulder (flex/ext)	0.80	0.59	0.82	0.88	0.76	0.91
SD	0.20	0.33	0.29	0.18	0.33	0.15
p-value	0.0038	0.047	0.040	0.004	0.032	0.0053
Shoulder (abd/add)	0.77	0.66	0.79	0.87	0.58	0.50
SD	0.21	0.43	0.29	0.14	0.50	0.46
p-value	< 0.001	0.03	0.04	< 0.001	0.02	0.04
Hip (flex/ext)	0.95	0.82	0.73	0.90	0.84	0.82
SD	0.06	0.22	0.35	0.08	0.28	0.25
p-value	< 0.001	0.0016	0.039	< 0.001	0.0077	0.016
Hip (abd/add)	0.63	0.84	0.71	0.74	0.93	0.89
SD	0.40	0.17	0.42	0.26	0.13	0.17
p-value	0.0325	0.0039	0.037	0.012	0.0072	0.0024
Knee (flex/ext)	0.99	0.99	0.88	0.99	0.80	0.96
SD	0.00	0.02	0.30	0.01	0.37	0.06
p-value	< 0.001	< 0.001	0.037	< 0.001	0.011	< 0.001

Table 4. RMSE comparison between PNS and VICON for raw values for all movements.

Joint Angle	Stationary walk	Distance walk	Stationary jog	Distance jog	Stationary wrist shot	Distance wrist shot
Elbow (flex/ext)	3.40	2.04	3.89	1.92	2.81	3.20
SD	2.15	1.48	2.96	1.00	2.18	1.75
Shoulder (flex/ext)	1.90	1.12	1.94	1.78	2.23	1.99
SD	0.80	0.65	1.53	1.16	1.97	1.12
Shoulder (abd/add)	7.14	5.36	5.97	5.70	11.85	15.15
SD	2.97	3.16	3.80	2.57	10.24	9.32
Hip (flex/ext)	2.78	2.63	2.91	2.76	1.83	3.58
SD	1.61	1.19	1.51	1.23	1.29	2.68
Hip (abd/add)	1.50	1.47	1.32	1.80	1.00	1.67
SD	0.95	1.29	0.85	0.95	0.62	0.86
Knee (flex/ext)	1.48	2.56	5.26	2.19	1.12	3.35
SD	0.56	1.16	9.13	1.27	0.81	2.23

Table 5a. Limits of agreement and mean bias for all stationary movements.

Joint Angle	Stationary walk Lower limit	Mean bias	Upper limit	Stationary jog Lower limit	Mean bias	Upper limit	Stationary wrist shot Lower limit	Mean bias	Upper limit
Elbow (flex/ext)	-42.55	-16.20	10.15	-68.01	-15.06	37.90	-40.53	-20.34	-0.15
correlation (heteroscedasticity)	$p < 0.001$		$r = -0.62$	$p < 0.001$		$r = -0.52$	$p < 0.001$		$r = -0.17$
coefficient of determination		$r^2 = 0.38$			$r^2 = 0.27$			$r^2 = 0.029$	
Shoulder (flex/ext)	-10.42	4.04	18.50	-11.87	2.61	17.09	-23.74	1.92	27.58
correlation (heteroscedasticity)	$p < 0.001$		$r = -0.53$	$p < 0.001$		$r = -0.39$	$p < 0.001$		$r = -0.39$
coefficient of determination		$r^2 = 0.28$			$r^2 = 0.15$			$r^2 = 0.16$	
Shoulder (abd/add)	-20.59	9.07	38.73	-15.39	12.16	39.72	-69.77	-7.52	54.72
correlation (heteroscedasticity)	$p < 0.001$		$r = -0.17$	$p < 0.001$		$r = -0.24$	$p < 0.001$		$r = -0.24$
coefficient of determination		$r^2 = 0.028$			$r^2 = 0.057$			$r^2 = 0.058$	
Hip (flex/ext)	-41.00	-0.56	39.88	-56.02	-5.21	45.59	-29.08	-4.11	20.85
correlation (heteroscedasticity)	$p < 0.001$		$r = -0.59$	$p < 0.001$		$r = -0.51$	$p < 0.001$		$r = -0.29$
coefficient of determination		$r^2 = 0.34$			$r^2 = 0.26$			$r^2 = 0.086$	
Hip (abd/add)	-13.56	-1.35	10.86	-12.62	-3.43	5.75	-19.26	-3.91	11.44
correlation (heteroscedasticity)	$p < 0.001$		$r = -0.40$	$p < 0.001$		$r = -0.31$	$p < 0.001$		$r = -0.60$
coefficient of determination		$r^2 = 0.16$			$r^2 = 0.096$			$r^2 = 0.36$	
Knee (flex/ext)	-19.88	-8.63	2.62	-43.36	-9.34	24.68	-16.49	-5.37	5.76
correlation (heteroscedasticity)	$p < 0.001$		$r = -0.50$	$p < 0.001$		$r = -0.16$	$p < 0.001$		$r = -0.16$
coefficient of determination		$r^2 = 0.25$			$r^2 = 0.025$			$r^2 = 0.025$	

Table 5b. Limits of agreement and mean bias for all movements with distance.

	Distance walk			Distance jog			Distance wrist shot		
**Joint Angle**	**Lower limit**	**Mean bias**	**Upper limit**	**Lower limit**	**Mean bias**	**Upper limit**	**Lower limit**	**Mean bias**	**Upper limit**
Elbow (flex/ext)	-35.47	-21.17	-6.88	-28.02	-12.22	3.57	-41.82	-20.62	0.57
correlation (heteroscedasticity)	*p* < 0.001		*r* = -0.23	*p* < 0.001		*r* = -0.43	*p* = 0.044		*r* = -0.014
coefficient of determination		r^2 = 0.056			r^2 = 0.19			r^2 = 0.00019	
Shoulder (flex/ext)	-3.49	7.26	18.01	-8.31	3.54	15.39	-26.81	-1.42	23.98
correlation (heteroscedasticity)	*p* < 0.001		*r* = -0.30	*p* < 0.001		*r* = -0.24	*p* < 0.001		*r* = -0.44
coefficient of determination		r^2 = 0.090			r^2 = 0.056			r^2 = 0.19	
Shoulder (abd/add)	-22.63	11.10	44.82	-22.24	9.80	41.85	-61.27	2.88	67.03
correlation (heteroscedasticity)	*p* < 0.001		*r* = -0.44	*p* < 0.001		*r* = -0.31	*p* < *0.001*		*r* = 0.31
coefficient of determination		r^2 = 0.19			r^2 = 0.099			r^2 = 0.10	
Hip (flex/ext)	-55.40	-11.18	33.05	-40.31	-5.24	29.82	-50.33	-7.42	35.49
correlation (heteroscedasticity)	*p* < 0.001		*r* = -0.69	*p* < 0.001		*r* = -0.70	*p* < 0.001		*r* = -0.56
coefficient of determination		r^2 = 0.47			r^2 = 0.49			r^2 = 0.32	
Hip (abd/add)	-12.88	-1.79	9.29	-12.54	-1.71	9.12	-16.17	-1.49	13.19
correlation (heteroscedasticity)	*p* < 0.001		*r* = -0.059	*p* < 0.001		*r* = -0.039	*p* < 0.001		*r* = -0.61
coefficent of determination		r^2 = 0.0034			r^2 = 0.15			r^2 = 0.37	
Knee (flex/ext)	-22.64	-7.72	7.19	-18.96	-6.64	5.69	-20.74	-7.53	5.67
correlation (heteroscedasticity)	*p* = 0.029		*r* = -0.14	*p* < 0.001		*r* = -0.29	*p* < 0.001		*r* = -0.14
coefficient of determination		r^2 = 0.018			r^2 = 0.087			r^2 = 0.019	

Table 6. LOA (%) comparison between PNS and VICON for raw and normalized values for all movements.

	Stationary walk		Distance walk		Stationary jog		Distance jog		Stationary wrist shot		Distance wrist shot	
**Joint Angle**	**Raw**	**Norm**	**Raw**	**Norm**	**Raw**	**Norm**	**Raw**	**Norm**	**Raw**	**Norm**	**Raw**	**Norm**
Elbow (flex/ext)	94.59	95.82	93.71	95.30	93.95	95.85	94.85	96.49	96.54	96.65	93.12	95.12
Shoulder (flex/ext)	95.27	95.91	96.23	96.80	94.50	96.15	96.22	96.46	96.57	96.77	92.68	95.16
Shoulder (abd/add)	95.41	95.36	95.26	95.48	93.70	95.85	95.59	96.70	94.81	95.48	92.61	94.47
Hip (flex/ext)	95.91	95.05	96.31	96.14	93.95	95.65	96.10	96.33	96.90	96.86	93.64	95.61
Hip (abd/add)	95.05	95.64	96.64	96.48	95.50	95.55	97.00	97.57	97.37	96.75	93.75	95.11
Knee (flex/ext)	95.00	95.10	94.38	95.45	94.70	95.05	94.86	96.26	97.48	96.89	94.40	95.83

Table 7. Range of joint angles tested for each movement.

	Stationary walk		Stationary jog		Stationary wrist shot		Distance walk		Distance jog		Distance wrist shot	
**Joint Angle**	**Lower**	**Upper**	**Lower**	**Upper**	**Lower**	**Upper**	**Lower**	**Upper**	**Lower**	**Upper**	**Lower**	**Upper**
Elbow (flex/ext)	38	170	39	154	60	165	105	170	47	168	54	158
Shoulder (flex/ext)	17	67	20	65	19	93	12	73	22	77	14	101
Shoulder (abd/add)	33	179	41	179	41	179	106	183	99	181	21	179
Hip (flex/ext)	101	171	86	165	92	172	45	175	94	175	87	175
Hip (abd/add)	82	107	83	109	76	133	35	109	82	113	74	126
Knee (flex/ext)	61	172	49	169	108	175	41	177	49	172	64	174

Discussion

The purpose of the study was to validate the PNS during full-body human motion capture in comparison to an accepted optoelectronic motion capture systems (i.e., VICON motion analysis system). To establish the validity of the PNS, elbow flexion/extension, shoulder flexion/extension, shoulder abduction/adduction, hip flexion/extension, hip abduction/adduction and knee flexion/extension angles were calculated from both systems and compared. These joint angles were analysed across movements such as stationary walk, distance walk, stationary jog, distance jog, stationary floorball wrist shot and moving wrist shot. Statistical analyses included RMSE, Pearson's R correlation and Bland-Altman analysis.

Overall, all joint angles across all movements had a RMSE of < 4°, except for shoulder abduction/adduction which had a large RMSE above 11° for both stationary and distance wrist shots. Pearson's R correlation also showed strong relationship for most joint angles except for shoulder flexion/extension during distance walk and hip abduction/adduction during stationary walk.

Specifically, values for knee flexion/extension across all movements were acceptable, having an average RMSE of 2.66° and a strong relationship with the highest average Pearson's R correlation value of 0.93 among all the other joints angles. There was overall good agreement between the two measurement systems for hip flexion/extension as well with an average Pearson's correlation value of 0.84 across all movements and all movements had a RMSE of < 4°. This indicates that the overall waveform for knee flexion/extension and hip flexion/extension is similar between the two measurement systems. These results were in accordance to results from other studies comparing IMU measurements against VICON motion capture systems. Nüesch, Roos (35) compared the sagittal plane ankle, knee and hip kinematics collected from an inertial sensor system and the VICON motion capture system. Kinematic data was collected during walking and running trials and reported that RMSE was found to be

below 5° for walking trials and below 8° for running trials across the different joint angles. In a study conducted by Bolink, Naisas (36), the use of an IMU was evaluated against the VICON motion capture system during three different activities of daily life: gait, sit-to-stand transfers and block step-up transfers. It was found that the IMU was a valid tool to measure dynamic pelvic angles with a strong Pearson's correlation ranging between 0.85 and 0.94 and RMSE values between 2.7° to 4.5°. Seel, Raisch (37) compared knee flexion/extension measurements between IMU and VICON motion capture systems during gait trials and found RMSE ranging from 1.62-3.3°. Even though Nüesch, Roos (35) reported a higher measurement error as a result of the increase in the speed of movement from walking to running, this difference was not evident in the current study possibly because walking and running speeds were self-selected.

In general, results for upper body joint angles were not as good as compared to lower body joint angles. This is in line with systematic reviews (38, 39) which reported that the large variability observed in the upper body measurements could be due to how functional upper body tasks usually involves complex movements within two-three axes. Even with this, the magnitude of error for elbow flexion/extension and shoulder flexion/extension were still comparable to those reported in previous studies. Pearson's R correlation (0.84 to 0.96) and RMSE (< 4°) for elbow flexion/extension measurements found in this study was similar to previous studies done (38, 40, 41). Shoulder (flex/ext) also had a moderate to strong Pearson's R correlation and an RMSE of < 3° across all movements which is within the range of error found in other studies (27, 38).

Results for shoulder abduction/adduction differed most from previous studies. Pearson' R correlation was found to range between moderate to strong (0.58 to 0.87) with lower values during stationary and distance wrist shots. RMSE was also higher than what was reported for other joints, ranging from 5.36° to 15.15°. Similar to Pearson's R correlation, RMSE values were also higher during stationary and distance wrist shots. Most studies have suggested that any RMSE up to 5° can be

regarded as reasonable and are precise enough for a system to be considered as a valid method for motion analysis in clinical settings. However, when errors are above 5°, it should raise concerns as it is considered large enough to mislead clinical interpretation (1, 36, 39, 42, 43). Therefore the use of raw shoulder abduction/adduction joint angles should be treated with caution, with the degree of acceptable limits directly dependent on the intended application (44).

Bland-Altman analysis showed that the mean systematic bias was below 10° for most joint angles with the exception of the elbow flexion/extension for all movements and shoulder abduction/adduction for distance walk (Table 4a and 4b). Bland-Altman plots for all raw and normalized joint angles can be found in Fig 3. Based on the recommendation of previous studies, a 10° lower or upper LOA was deemed acceptable for occupational biomechanics applications (27, 45, 46). Therefore joint angles obtained from the PNS seems satisfactory except for elbow flexion/extension and shoulder abduction/adduction for which data should be handled with discretion especially during dynamic movements when absolute measurement of joint angles is required. Results showed significant correlation between the mean joint angle value and the difference between the two measurements system for almost all the joints and across movements. Therefore, there is a possibility of heteroscedasticity (34) where the amount of error from the PNS might increase as the measured joint angle value increases. Significant correlation was found even with a small coefficient of determination (r^2) between the mean joint angles and difference between the two measurements. The observed significance despite a very small r^2 is most probably due to the large sample size (above 19000 data points). However, validation is still confirmed for the range of movement that was investigated in this study (see Table 7), but caution should be applied when analyzing larger joint angles (when higher values are possible). OK, good!

what about hip ab/ad?

When using normalized and raw joint angles for comparison between the two systems, LOA (%) results were consistently higher for normalized values (range from 94.47% to 97.57%) as compared to

raw joint angles (range from 92.49% to 97.52%). In addition, it was observed that joint angle wave characteristics were more similar when using normalized rather than the raw joint angles. The normalization process of the joint angles managed to preserve the waveform characteristics (e.g., peak joint moment) and reduce the variance between the two systems. As such, it is suggested that when using the PNS, the analysis of normalized joint angles is preferred instead of the raw joint angles as the PNS is better at detecting changes in movement patterns rather than to approximate the absolute joint angles.

Limitations

A limitation of the current study is the small collection time frame for each trial. It has been reported in previous studies that IMUs accuracy differed accordingly to the type of tasks being performed and is affected by the duration, complexity and speed of the task performed (27, 35, 38, 47-50). Accuracy of IMUs is also affected by time of the recorded task. Its accuracy decreases over time as a result of drift which continually increases and the amount of error is compounded over time (50, 51). Drift occurs due to the magnetic perturbations in the environment around the sensors and also the ability of the sensors to locate and track the same initial frames (52). The amount of drift is dependent on the system and the direction of movement, with accuracy decreasing more for movements that occur within 3-axes over a sustained period of time (52). Data included in the current study was collected within 20-30 seconds before the next calibration. Therefore, future studies could include longer trials to detect how much the PNS drifts over time and its resilience against magnetic perturbation in order to provide more information on its accuracy and if any correction is needed in the algorithms.

Previous studies have found that the accuracy of IMUs is negatively influenced by speed (53). Larger RMSE was reported by Nüesch, Roos (35) during running as compared to walking on a treadmill,

with errors of < 8° and < 5° respectively. Similarly, Cooper G., Sheret I. (47) found a RMSE of < 4° during running and < 1° of the knee during walking. However, the effect of speed on the accuracy of the PNS was not as evident as in other studies. Comparisons between walking and jogging tasks did not elicit a significant change in the results. One possible reason could be that the walking and jogging speeds were self-selected and not controlled, therefore the walking and jogging speeds might not have been significantly different enough to result in a difference. This result was similar to the study conducted by Sers, Forrester (1) where no obvious difference was detected with a change in movement speeds as speeds were also self-selected. Therefore, future studies could also analyse the accuracy of the PNS with movements at different controlled speeds.

and

Conclusion & recommendations

The validity of the PNS was tested against the VICON motion analysis system by comparing joint angle measurements obtained by the two systems during a series of full-body human movements. In general, the PNS performed well against the VICON motion analysis system and most joint comparisons were similar to that of what was reported in previous studies, with the exception of elbow flexion/extension and shoulder abduction/adduction during the stationary and distance wrist shots. and hip ab/ad

With reference to Bland-Altman analysis, most of the joint angles were found to have been overestimated by the PNS with the exception of the shoulder flexion/extension and shoulder abduction/adduction. Mean bias for most joint angles were within acceptable recommendations from previous studies (27, 45, 46). LOA range when using raw joint angles was larger than what is recommended for biomechanics applications (27, 45, 46). Thus, caution should be applied when using raw joint angle measurements obtained from the PNS for analysis. In addition, validation of the PNS is confirmed for the range of movements that were investigated in this study and caution should also be applied when analyzing any joint angles beyond this range. When using normalized joint angles for

comparisons, LOA (%) values were consistently better as compared to when raw joint angles were used. In conclusion, results suggests that the PNS could be more suited to analyse movement patterns through the use of normalized rather than raw joint angles and that researchers could consider the use of the PNS when doing so.

References

1. Sers R, Forrester S, Moss E, Ward S, Ma J, Zecca M. Validity of the Perception Neuron inertial motion capture system for upper body motion analysis. Measurement. 2020;149.

2. Colyer AL, Evans M, Cosker DP, Saldo A. A review of the evolution of vision-based motion analysis and the integration of advanced computer vision methods towards developing a markerless system. Sports Medicine- Open 2018;4(24).

3. Van der Kruk E, Reijne MM. Accuracy of human motion capture systems for sport applications; state-of-the-art review. European Journal of Sport Science. 2018;18(6).

4. Beange KHE, Chan ADC, Graham RB. Evaluation of wearable IMU performance for orientation estimation and motion tracking. IEEE International Symposium on Medical Measurements and Applications (MeMeA)2018.

5. Sessa S, Zecca M, Lin Z, Bartolomeo L, Ishii H, Takanishi A. A methodology for the performance evaluation of inertial measurement units. Journal of Intelligent & Robotic Systems. 2012;71(2):143-57.

6. Topley M, Richards JG. A comparison of currently available optoelectronic motion capture systems. Journal of biomechanics. 2020;106.

7. Hemming R, Sheeran L, van Deursen R, Sparkes V. Non-specific chronic low back pain: differences in spinal kinematics in subgroups during functional tasks. European Spine Journal 2018;27:163-70.

8. Frost DM, Beach TAC, Callaghan JP, McGill SM. Using the functional movement screen™ to evaluate the effectiveness of training. Journal of Strength and Conditioning Research. 2012;26(6):1620-30.

9. Lazzaeri M, Kayser B, Armand S. Kinematic predictors of wrist shot success in floorball/unihockey from two different feet positions. Journal of Sports Sciences. 2016;34(21):2087-94.

10. Chiu LZF, Bryanton MA, Moolyk AM. Proximal to distal sequencing in vertical jumping with and without arm swing. Journal of Strength and Conditioning Research. 2014;28(5):1195-202.

11. Zhang Y, Ma Y, Liu G. Lumbar spinal loading during bowling in cricket: a kinetic analysis using a musculoskeletal modelling approach. Journal of Sports Sciences. 2016;34(11):1030-5.

12. Bisesti BA, Lawrence MA, Koch AJ, Carlson LA. Comparison of knee moments and landing patterns during a lateral cutting maneuver: Shod vs. Barefoot. . Journal of strength and conditioning research. 2015;29(11):3075–8.

13. Hellmers S, Fudickar S, Lange E, Lins C, Hein A. Validation of a motion capture suit for clinical gait analysis. The 11th EAI International Conference on Pervasive Computing Technologies for Healthcare2017.

14. Pueo B, Jimenez-Olmedo J. Application of motion capture technology for sport performance analysis. Retos. 2017;32:241-7.

15. Chardonnens J, Favre J, Cuendet F, Gremion G, Aminian K. A system to measure the kinematics during the entire ski jump sequence using inertial sensors. Journal of biomechanics. 2013;46(1):56-62.

16. Sharif Bidabidi S, Murray I, Lee GYF. The application of inertial measurements unit for the clinical evaluation and assessment of gait events. Journal of Medical Engineering & Technology. 2017;41(8):612-22.

17. Walker C, Sinclair P, Graham K, Cobley S. The validation and application of inertial measurement units to springboard diving. Sports Biomechanics. 2016;16(4):185-500.

18. Robert-Lachaine X, Mecheri H, Larue C, Plamondon A. Validation of inertial measurement units with an optoelectronic system for whole‐body motion analysis. Medical & Biological Engineering & Computing. 2017;55(4):609-19.

19. Wirth MA, Fischer G, Verdú J, Reissner L, Balocco S, Calcagni M. Comparison of a new inertial sensor based system with an optoelectronic motion capture system for motion analysis of healthy human wrist joints. Sensors. 2019;19(23):5297.

20. Filippeschi A, Schmitz N, Miezal M, Bleser G, Ruffaldi E, Stricker D. Survey of motion tracking methods based on inertial sensors: A focus on upper limb human motion. Sensors. 2017;17(6):1257.

21. Bosch S, Shoaib M, Geerlings S, Buit L, Meratnia N, Havinga P. Analysis of indoor rowing motion using wearable inertial sensors. BODYNETS. 2015.

22. Shepherd JB, Giblin G, Pepping GJ, Thiel D, Rowlands D. Development and validation of a single wrist mounted inertial sensor for biomechanical performance analysis of an elite netball shot. IEEE Sens. 2017;1(5):1-4.

23. Whelan D, O'Reilly M, Huang B, Giggins O, Kechadi T, Caulfield B. Leveraging IMU data for accurate exercise performance classification and musculoskeletal injury risk screening. 38th Annual International Conference of the IEEE Engineering in Medicine and Biology Society (EMBC) 2016.

24. Makhni EC, Lizzio VA, Meta F, Stephens JP, Okoroha KR, Moutzouros V. Assessment of elbow torque and other parameters during the pitching motion: Comparison of fastball, curveball, and change-up. Arthroscopy: The journal of arthroscopic & related surgery. 2018;34(3):816-22.

25. Rawashdeh S, Rafeldt D, Uhl T. Wearable imu for shoulder injury prevention in overhead sports. Sensors. 2016;16(11):1847.

26. Wang Y, Zhao Y, Chan RHM, Li WJ. Volleyball skill assessment using a single wearable micro inertial measurement unit at wrist. IEEE Access. 2018;6(13758-13765).

27. Robert-Lachaine X, Mecheri H, Larue C, Plamondon A. Validation of inertial measurement units with an optoelectronic system for whole-body motion analysis. Medical & Biological Engineering & Computing 2017;55(4):609-19.

28. Adesida Y, Papi E, McGregor AH. Exploring the role of wearable technology in sport kinematics and kinetics: A systematic review. Sensors. 2019;19(7):1597.

29. Cho YS, Jang SH, Cho JS, Kim MJ, Lee HD, Lee SY, et al. Evaluation of validity and reliability of inertial measurement unit-based gait analysis systems. Annals of rehabilitation medicine. 2018;42(6):872-83.

30. Lee MCYL, Chow JY, Komar J, Tan CWK, Button C. Nonlinear pedagogy: An effective approach to cater for individual differences in learning a sports skill. PLoS ONE. 2014;9(8).

31. Mould RF. Introductory Medical Statistics. 3rd ed. London, United Kingdom: Taylor & Francis Ltd; 1998.

32. Bland JM. Correction to section “Measuring agreement using repeated measurements” in Bland and Altman (1986). Statistical Methods in Medical Research. 1986:21-3.

33. Bland JM, Altman DG. Statistical methods for assessing agreement between two methods of clinical measurement. Lancet. 1986;1(8476):307–10. .

34. Atkinson G, Nevill AM. Statistical methods for essassing measurement error (reliability) in variables relevant to sports medicine. Sports Medicine. 1998;26:217-38.

35. Nüesch C, Roos E, Pagenstert G, Mündermann A. Measuring joint kinematics of treadmill walking and running: Comparison between an inertial sensor based system and a camera-based system. Journal of Biomechanics. 2017;57:32-8.

36. Bolink SAAN, Naisas H, Senden R, Essers H, Heyligers IC, Meijer K, et al. Validity of an inertial measurement unit to assess pelvic orientation angles during gait, sit–stand transfers and step-up

transfers: Comparison with an optoelectronic motion capture system. Medical Engineering & Physics. 2016;38(3):225.

37. Seel T, Raisch J, Schauer T. IMU based joint angle measurement for gait analysis. Sensors. 2014;14(4):6891-909.

38. Poitras I, Dupuis F, Bielmann M, Campeau-Lecours A, Mercier C, Bouyer L, et al. Validity and reliability of wearable sensors for joint angle estimation: A systematic review. Sensors. 2019;19(7):1555.

39. Walmsley CP, Williams, S. A., Grisbrook, T., Elliott, C., Imms, C., & Campbell, A. Measurement of upper limb range of motion using wearable sensors: A systematic review. . Sports Medicine. 2008;4(1).

40. El-Gohary M, Holmstrom L, Huisinga J, King E, McNames J, Horak F, editors. Upper limb joint angle tracking with inertial sensors. Annual International Conference of the IEEE Engineering in Medicine and Biology Society (EMBC); 2011; Boston, MA, USA.

41. Morrow MMB, Lowndes B, Fortune E, Kaufman KR, Hallbeck MS. Validation of inertial measurement units for upper body kinematics. Journal of Applied Biomechanics. 2017;33(3):227-32.

42. McGinley JL, Baker R, Wolfe R, Morris ME. The reliability of three-dimensional kinematic gait measurements: A systematic review. Gait & Posture. 2009;29:360-9.

43. Cuesta-Vargas AI, Galán-Mercant A, Williams JM. The use of inertial sensors system for human motion analysis. Phys Ther Rev 2010;15(6):462-73.

44. Teufl W, Miezal M, Taetz B, Fröhlich M, Bleser G. Validity of inertial sensor based 3D joint kinematics of static and dynamic sport and physiotherapy specific movements. PLOS ONE. 2019;14(2).

45. El-Zayat B, Efe T, Heidrich A, Anetsmann R, Timmesfeld N, Fuchs-Winkelmann S, et al. Objective assessment, repeatability, and agreement of shoulder ROM with a 3D gyroscope. BMC Musculoskeletal Disorders, 14. 2013;14:72-.

46. Schiefer C, Rolf PE, Hermanns I, Thomas K, Ochsmann E, Larue C, et al. Optimization of inertial sensor-based motion capturing for magnetically distorted field applications Journal of Biomechanical engineering 2014;136.

47. Cooper G., Sheret I., McMillian L., Siliverdis K., Sha N., Hodgins D., et al. Inertial sensor-based knee flexion/extension angle estimation. Journal of Biomechanics 2009;42(16):2678-85.

48. Dejnabadi H, Jolles BM, Aminian K. A new approach to accurate measurement of uniaxial joint angles based on a combination of accelerometers and gyroscopes. IEEE Transactions on Biomedical Engineering. 2005;52(8):1478-84.

49. Papi E, Osei-Kuffour D, Chen Y-MA, McGregor AH. Use of wearable technology for performance assessment: A validation study. Medical Engineering & Physics. 2015;37(7):698-704.

50. Martori AL. A wearable motion analysis system to evaluate gait deviations. South Florida: University of South Florida; 2013.

51. Kim S, Nussbaum MA. Performance evaluation of a wearable inertial motion capture system for capturing physical exposures during manual material handling tasks. Ergonomics. 2013;56(2):314-26.

52. Lebel K, Boissy P, Hamel M, Duval C. Inertial measures of motion for clinical biomechanics: Comparative assessment of accuracy under controlled conditions – changes in accuracy over time. PLOS ONE. 2015;10(3).

53. Blair S, Duthie G, Robertson S, Hopkins W, Ball K. Concurrent validation of an inertial measurement system to quantify kicking biomechanics in four football codes. Journal of Biomechanics. 2018;73:24-32.

Supporting information

S1 Fig 1. Perception Neuron IMU sensor configuration.

S1 Fig 2. Participant set-up with both PNS and VICON markers front and back. The red squares indicates the PN sensor positions.

S1 Fig 3. Bland-Altman plot of agreement for hip flexion/extension for distance wrist shot using (a) raw joint angles (b) normalized joint angles for all participants. Solid horizontal lines represent the mean difference and the dashed horizontal lines represents the 95% limits of agreement (± 1.96 SD).

S1 Fig 4. Hip flexion/extension angles during a stationary wrist shot using raw joint angle values (a) and Hip flexion/extension angles during a stationary wrist shot using normalized joint angle values (b). Data shown is from one trial from one participant.

Click here to access/download;Figure;Fig1.tif

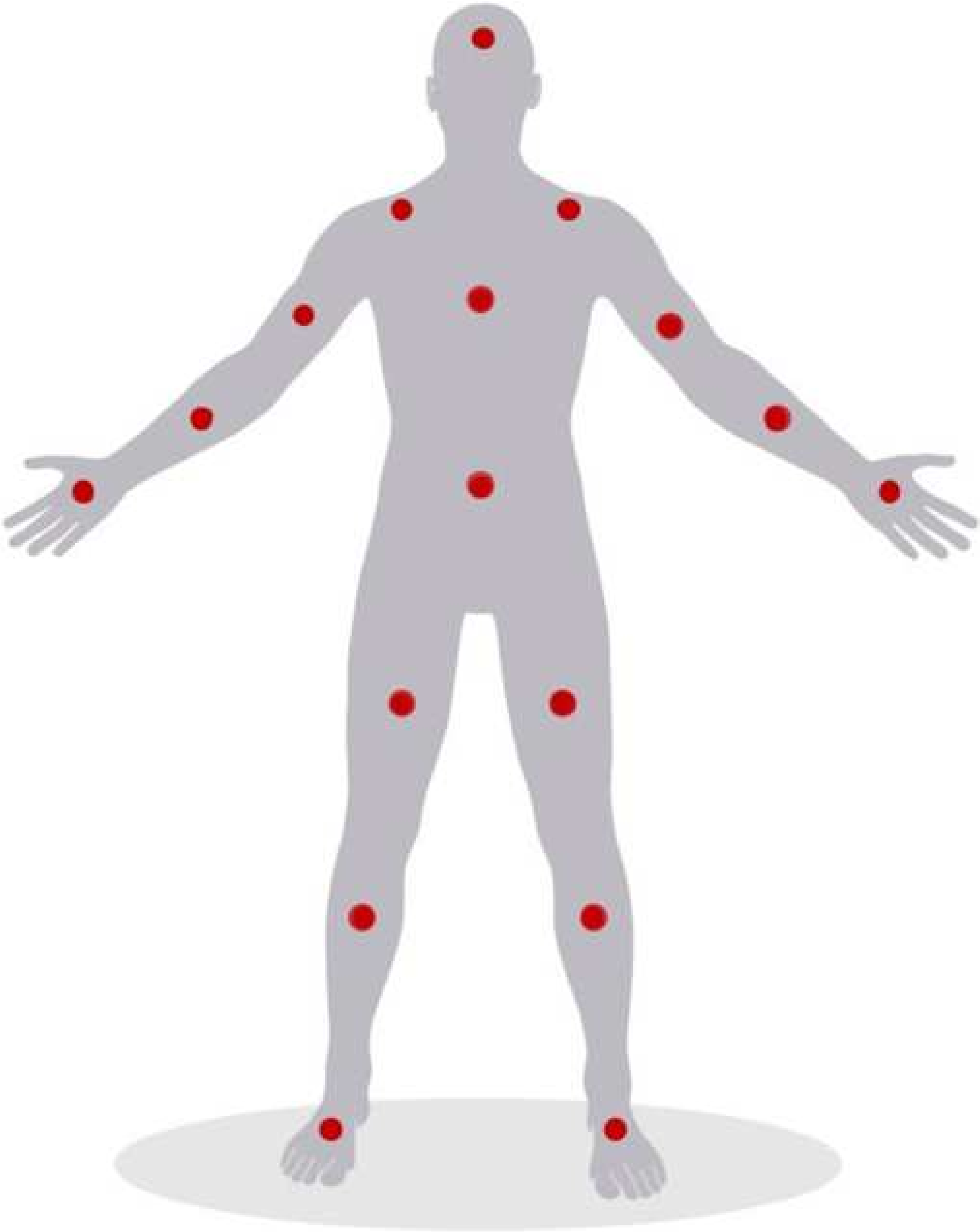

Figure Click here to access/download;Figure;Fig2.tif

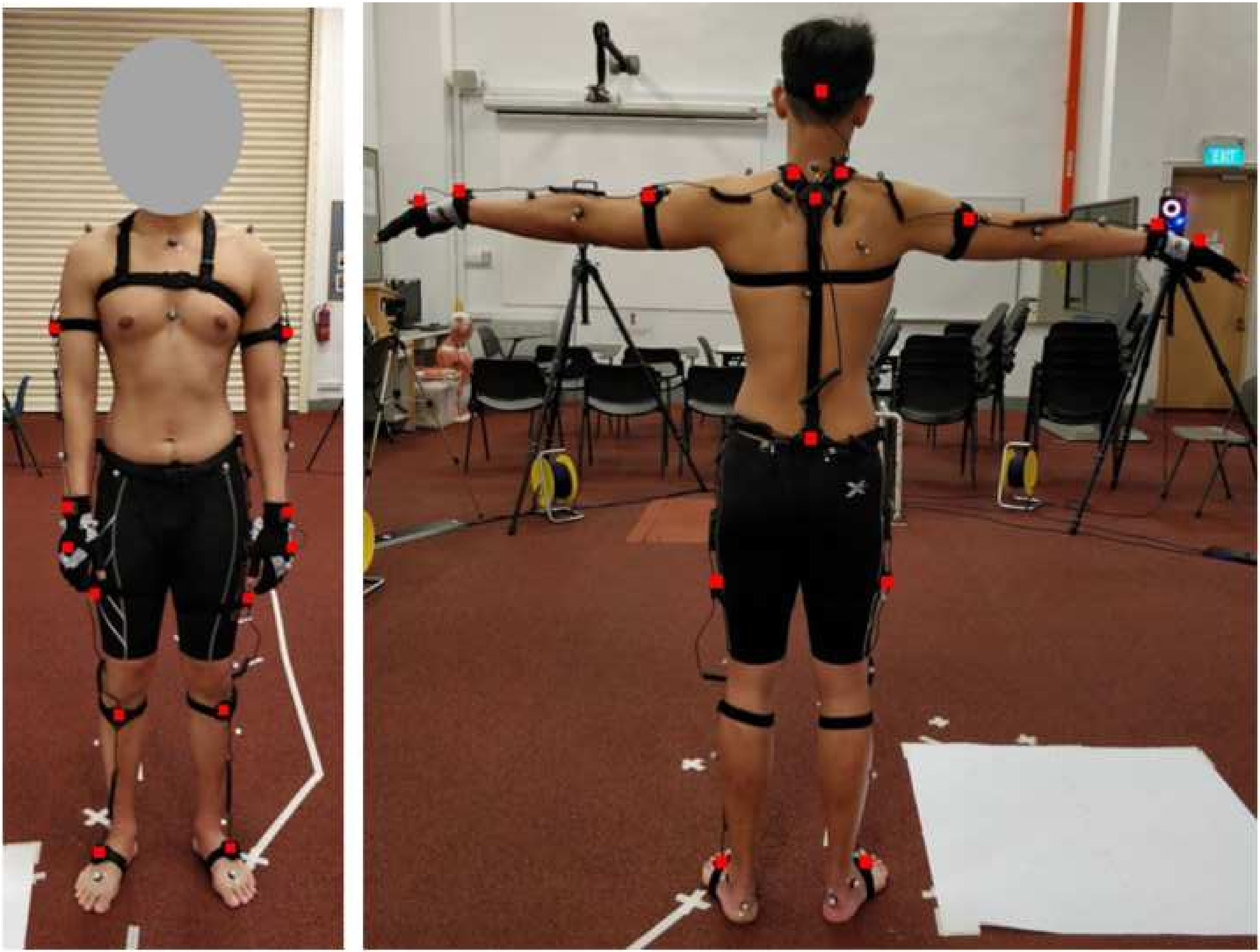

 Click here to access/download;Figure;Fig3.tif

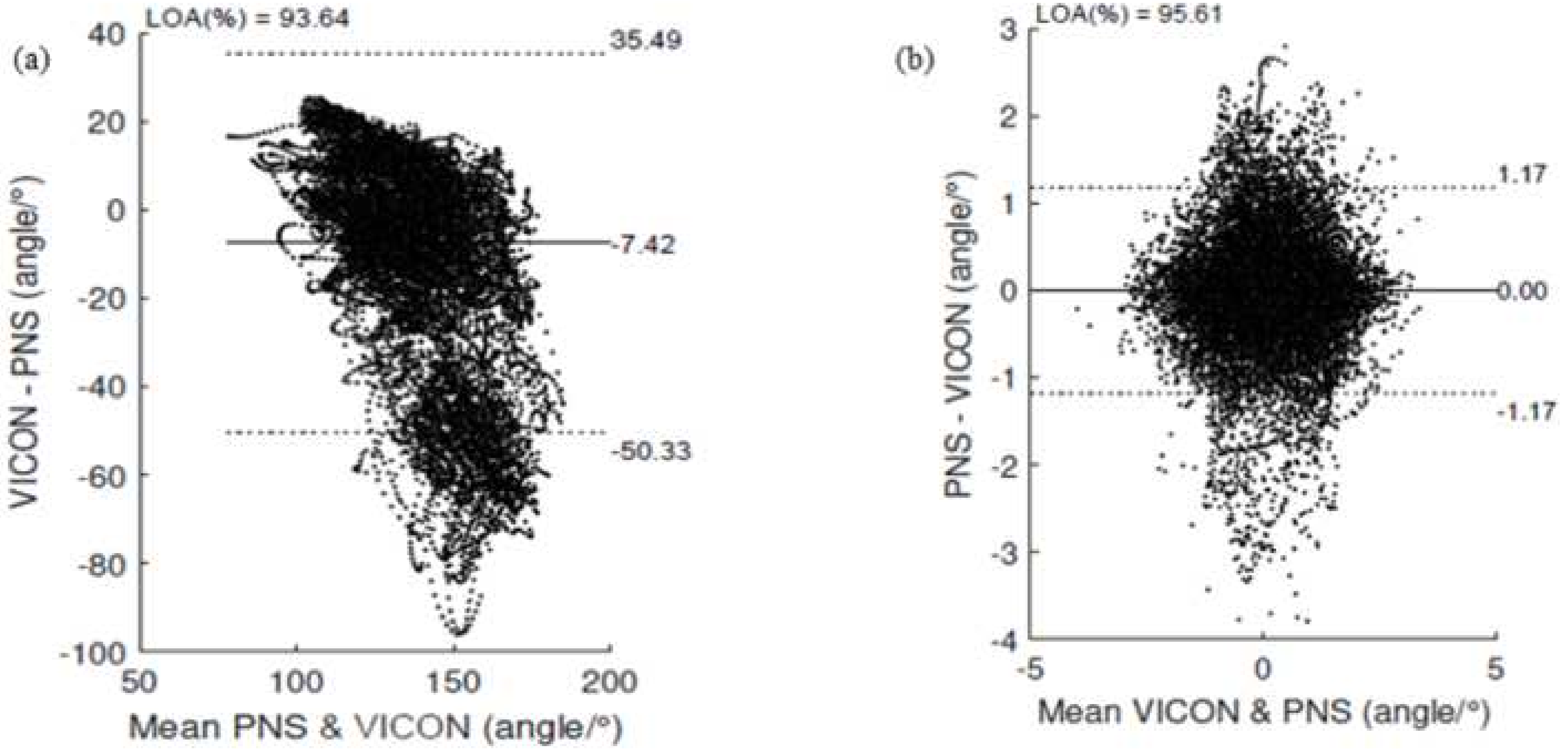

Figure

Click here to access/download;Figure;Fig4.tif

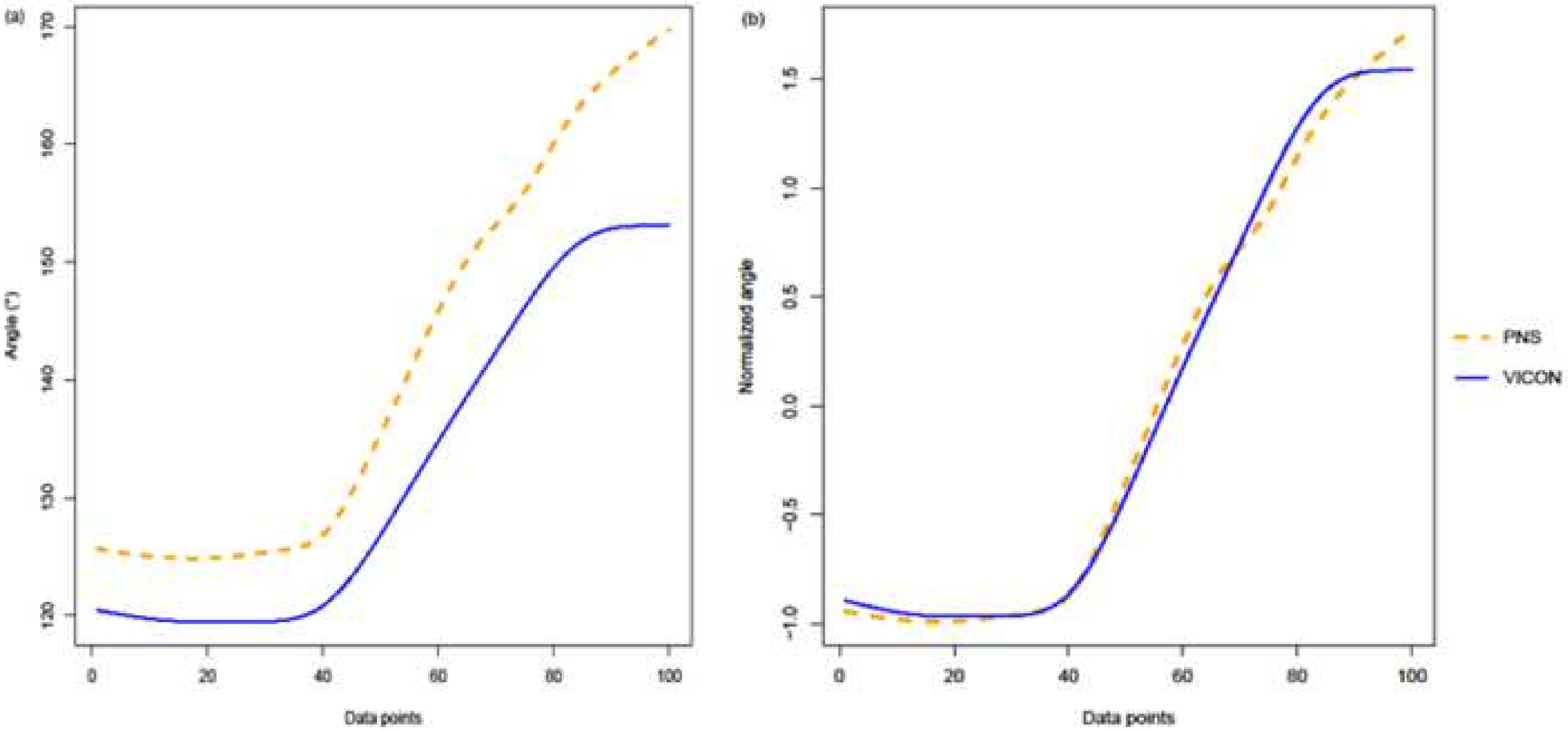

Supporting information

Click here to access/download
Supporting Information
Bland altman plots.docx
