# Supplementary material for: Validation of the Perception Neuron system for full-body motion capture
Source: PLoS One. 2022 Jan 21;17(1):e0262730. doi: 10.1371/journal.pone.0262730 (PMC8782534; doi:10.1371/journal.pone.0262730)
Supplement: Attachment — Submitted filename: Response to reviewers.docx [file pone.0262730.s003.docx]

Thank you for the positive review and constructive comments. Below, we highlight the revisions made to enhance the manuscript.

| Pg 3, row 52.  Replace method with methods. | This has been revised. See pg 3, row 52. |
| --- | --- |
| Pg 3, row 53.  Replace up to < with less than. | This has been revised. See pg 3, row 53 to 54. |
| Pg 3, row 60.  Insert comma. | This has been revised. See pg 3, row 60. |
| Pg 3, row 64.  Replace its with their. | This has been revised. See pg 3, row 64. |
| Pg 4, row 72-77  Comment: Although true, most systems have enough cameras that this is not a problem… For most lab collections obstruction is not an issue. Is there a specific application you have in mind where it is? | The purpose of the study was to investigate and potentially provide evidence for researchers that the PNS could be a viable tool to collect motion capture data in a more representative environment outside of a lab setting.  This is especially so when it is not feasible to set up an optical based system. For example in situations where there is a need to analyse movement over a larger area, if an optical based system is used, a greater number of cameras would be required to cover this increase in volume. In this case, the PNS could be used to overcome the limitations of the lab space and the number of cameras needed. |
| Pg 4, row 89-90.  Comment: Is this compared to earlier IMUs? What’s the accuracy? You cited the accuracy of the optical mocap systems earlier. | Yes, this is in comparison with earlier IMUs. As the accuracy of the sensors are highly dependent on the sensor specifications, software algorithms, movement type and joint angle analysed there is a large range for this value. Therefore, no exact value was indicated in this section. This was highlighted on pg 5, row 106 to 109. |
| Pg 6, row 124.  Comment: Uncommon word, bias is probably preferable. | This has been revised. See pg 6, row 124. |
| Pg 6, row 133.  Insert comma. | This has been revised. See pg 6, row 133. |
| Pg 6, row 138.  Comment: Be consistent with the use of PNS. | This has been revised. Has been changed to PNS for these instances:  Pg 6, row 137; pg 8, row 175: pg 9, row 180; pg 11, row 206. |
| Pg 7, row 149.  Comment: Spell out the number. | This has been revised. See pg 7, row 149. |
| Pg 7, row 152.  Change illnesses to illness. | This has been revised. See pg 7, row 152. |
| Pg 7, row 152.  Change diseases to disease. | This has been revised. See pg 7, row 153. |
| Pg 7, row 159.  Change from the most widely used to a widely used system? | This has been revised. See pg 7 row 159. |
| Pg 7, row 160.  Comment: Was the statement “The VICON optoelectronic motion analysis system was chosen as it is a widely used system and is considered as the current laboratory gold-standard with a high accuracy during dynamic trial.” the result of Topley & Richards (2020)? | Topley & Richards (2020) did not specifically mention that the VICON was a widely used system or that it was considered as the current lab gold standard. It did indicate the high accuracy of the VICON and other camera systems, however testing was conducted using the surface of a rigid aluminum arm that rotates. Therefore, this reference was not added at the end of the sentence since it did not support the statement. |
| Pg 7, row 161.  Spell out the number in the sentence. | This has been revised. See pg 7, row 161. |
| Pg 7, row 163.  Comment: Does the suit increases or decrease the soft tissue artifact from the markers? Is this a source of uncertainty? | The amount of error resulting from this was not accounted for in the current study. Therefore, It has been included as a limitation, see pg 24, row 374 to 377. |
| Pg 8, row 164.  Comment: Does the USB connection affect peoples’ movement as old EMG system wires used to? | The wires connecting between each sensor uses a coiled design, so it allows participants to stretch and move through the range of movement freely when necessary but at the same time is still neatly kept which prevents any excessive wiring from dangling around to potentially impede movement. The system connects through a single wire to the laptop and this wire can be placed to face different directions and be shifted out of the way to accommodate various movements. |
| Pg 10, row 190.  Change Natrick to Natick. | This has been revised. See pg 10, row 190. |
| Pg 10, 202.  Comment: Reference? Is this Cohen? | The citation has been added. See pg 10, row 203. |
| Pg 11 row, 206.  Comment: Change X to X(t) and Y to Y(t). Indicate that these are time dependent and that you are summing over time. | This has been revised. See pg 11, row 206 to 207. |
| Pg 12, row 227.  Comment: Distance wrist shot shoulder also not a strong correlation. | This has been added. See pg 12, row 229 to 230. |
| Pg 12, row 238.  Comment: This is only for flexion/extension for the wrist shot. | This has been revised to state that the figure is only indicative for hip (flexion/extension) during distance wrist shot. See pg 12, row 241 to 243. |
| Pg 13, row 244.  Comment: How were data normalized? | Data was normalized using z-score transformation. Details have been added to pg 11, row 218 to 221. |
| Pg 22.  Comment: What about hip ab/ad? | The mean systematic bias for hip flexion/extension during distance walk was found to be -11.18°. This has been included. Hip abduction/adduction was -1.35 for stationary walk and -1.79° for distance walk.  See pg 22, row 323 to 324, pg 323 row 328 to 329, and pg 24 row 383 to 384. |
| Pg 24.  Change & to and. | This has been revised. See pg 24, row 377 and pg 24, row 378. |
