# Supplementary material for: Validation of the Perception Neuron system for full-body motion capture
Source: PLoS One. 2022 Jan 21;17(1):e0262730. doi: 10.1371/journal.pone.0262730 (PMC8782534; doi:10.1371/journal.pone.0262730)
Supplement: Attachment — Submitted filename: Response to Reviewers.docx [file pone.0262730.s004.docx]

**Responses to Reviewers**

Thank you for the positive review and constructive comments. Below, we highlight the revisions made to enhance the manuscript.

| **Reviewer 1 comments** |  |
| --- | --- |
| It seems that the output from each system was filtered in the exact same way (page 10, lines 195-197). How did the authors determine that the frequency content to be filtered from both systems for each activity was identical and, therefore, presumably they used the same filtering process and parameters? Data need to be provided towards this end. If this comment is not correct, then how each signal output from each device for each activity was filtered and why? This information is directly related to the quality of the data output being compared. | Both systems were set to a recording speed of 120 Hz (see page 7, row 163). In addition, all trials were time normalized to 100 data points before comparisons were made. More details have been added to page 10, row 208 – 209. |
| The authors have used the Plug-In-Gait marker set to compute the kinematics for the activities they studied (page 7, lines 161-162). The Plug-In-Gait marker set, however, is tied to several assumptions tied to the Plug-In-Gait computational model. It appears, however, that the authors did not use the Plug-In-Gait model to compute the kinematics of interest (page 10, lines 187-191). Why did the authors use this marker set model? The marker model itself does not ensure 3D segment definition by 3 marker non-colinearity per segment.  Therefore, what assumptions did the authors use for the purposes of 3D segment definition (especially for the femur and tibia/fibula) and corresponding 3D motion computation? This information is also directly related to the quality of the data output being compared. | The Plug-In-Gait model was initially chosen as it was found to be a commonly used model for clinical gait analysis (Kainz et al., 2017).  It was eventually not used to compute the kinematics of interest (calculations were not computed on the VICON system itself), however the market set that was included in that model was still used for calculations on a separate code. This was done to ensure that the kinematics obtained from both systems were calculated on the same basis so that they were comparable.  Kainz, H., Graham, D., Edwards, J., Walsh, H. P. J., Maine, S., Boyd, R. N., Lloyd, D. G., Modenese, L., & Carty, C. P. (2017). Reliability of four models for clinical gait analysis. *Gait & Posture*, *54*, 325–331. https://doi.org/10.1016/j.gaitpost.2017.04.001 |
| Observing Figure 2, it appears that pelvis is defined by the 2 ASIS markers and the 2 PSIS markers. These markers appear to have been placed on the shorts/trunks of the subject, presumably directly over the specific anatomical landmarks (it is not clear, but it appears that a mid-thigh marker is also placed on the subject’s trunks). How did the authors made certain that all these markers on the trunks remained over the anatomical landmarks of interest throughout the duration of all activities after each calibration? This information is also directly related to the quality of the data output being compared. | Compression garments were used during the data collection process to minimize the marker movements whilst still allowing participants to be covered. It was chosen as it had previously reported that markers placed over compression garments demonstrate low variance in movement compared to the markers placed on the skin (Mills et al., 2011).  Furthermore, visual checks were also done during the collection process to ensure that the markers remained over the anatomical landmarks.  Mills, C., Scurr, J., & Wood, L. (2011). A protocol for monitoring soft tissue motion under compression garments during drop landings. *Journal of Biomechanics*, *44*(9), 1821–1823. https://doi.org/10.1016/j.jbiomech.2011.04.019 |
| Observing Figure 2, it appears that the mid-thoracic marker (typically placed on the 10^th^ thoracic spinous process) is placed neither on the 10^th^ thoracic spinous process, nor in line with the thoracic spinous processes. This would create a trunk orientation offset. How has such an offset been accounted for during the kinematic computation of the shoulder motion, which, presumably is measured relative to the trunk? | The T10 marker was shifted up to the same level as the sternum marker to make it easier to find the location. This was also possible as previously mentioned, the Plug-in-gait model was eventually not used to compute the kinematics in order to ensure that the kinematics from both systems were calculated on a similar basis. This detail was included in the revised submission on page 8, row 169-171.  The shifted T10 marker was also not utilized in the calculation of both shoulder flexion/extension and shoulder abduction/adduction. |
| Transverse plane data are very important, especially when considering injury prevention (in sports and occupational biomechanics) and in the clinical decision-making process. Given the ambitions of the authors for the PNS outlined in the introduction, the authors need to provide transverse data output comparisons between the 2 systems. | The focus of the current study was to look at the joint angle data for a future study that looked at the specific joint angles as mentioned in the current study. Therefore, transverse plane data was not included in the comparisons. This will be added in as a limitation of the current study for future studies to look into data within the transverse plane on page 25, row 394-396. |
| Observing Figures 1 and 2, it appears that PNS can provide ankle-related kinematics. Given the importance of ankle kinematics, especially for all lower extremity activities (in walking gait approximately 80% of the ambulatory power generation is related to the ankle), and given the desire of the authors to involve functional activities in this study, ankle kinematic output comparisons need to be made, even if only for the sagital plane and consistently with the study of Nuesch and Ross. | In the previously submitted version, the ankle kinematics were not included in the data analysis. Ankle data has been calculated and included in the resubmission. Additions were made to:   - Page 10, row 203 - Page 13, row 241-242 - Page 13, row 246-247 - Page 13, row 251-252 - Page 13, row 254-255 - Page 14, row 266-267 - Tables on page 15-20 - Page 21, row 294-295 - Page 21, row 299-230 - Page 21, row 304-305 - Page 21, row 310-311 - Page 23, row 348-349 - Page 23, row 353 - Page 26, row 412 - Bland-altman plots |
| From Tables 3 and 4 it appears that for the same activity the r and RMSE increased and decreased, respectively, for most activities as a function of increasing speed. The authors suggest that this may be related to subjects performing these activities at self-selected speeds. Although performing an activity at a self-selected speed, indeed, has been found to decrease the within trial or within condition/speed variability, the mere increase in velocity results in higher kinematic variability in VICON even from a motion artifact point of view. Therefore, the authors’ argument of self-selected speed is ambiguous. On the contrary, this suggests that either PNS’s performance for activities that are static or quasi static is substandard or the earlier arguments regarding the filtering process need to be given serious consideration. | The reason for the self-selected speed, rather than controlling that aspect, was to ensure that the movement tested was representative to how it is executed in a typical real-life performance setting. Instead of testing the system at specific speeds, the aim of the study was really to look at its ability to perform during functional movements.  The value of allowing movement to be demonstrated in a representative learning setting is that it facilitates important dynamic interactions between performer, task and environment constraints thus ensuring that close couplings between information and movement are preserved (Button et al., 2020; Chow, 2013; Pinder et al., 2011). When information present during training or practice simulates that of the performance setting, it allows the learner to develop the capacity to explore and create functional and individualized performance solutions (Button et al., 2020; Chow, 2013; Pinder et al., 2011).  Button, C., Seifert, L., Chow, J. Y., Araújo, D., & Davids, K. (2020). *Dynamics of skill acquisition: An ecological dynamics approach* (2nd ed.). Human Kinetics.  Chow, J. Y. (2013). Nonlinear learning underpinning pedagogy: Evidence, challenges, and implications. *Quest*, *65*(4), 469–484. https://doi.org/10.1080/00336297.2013.807746  Pinder, R. A., Davids, K., Renshaw, I., & Araújo, D. (2011). Representative learning design and functionality of research and practice in sport. *Journal of Sport and Exercise Psychology*, *33*(1), 146–155. https://doi.org/10.1123/jsep.33.1.146 |
| What is the normalization process implemented in the Bland altman plots? | Normalization of joint angle data was conducted using z-score transformation as indicated on page 12, row 232 - 235. |

| **Reviewer 2 comments** |  |
| --- | --- |
| I originally had a number of comments on the rewrite for lines 68-77. In particular these were regarding the ease of setup and the occlusion of markers. I suggest that your comments below address my concerns and that this wording, or something similar should be in the actual paper not just in the response should the editors permit the increase in word count.  "The purpose of the study was to investigate and potentially provide evidence for researchers that the PNS could be a viable tool to collect motion capture data in a more representative environment outside of a lab setting."  "This is especially so when it is not feasible to set up an optical based system. For example in situations where there is a need to analyse movement over a larger area, if an optical based system is used, a greater number of cameras would be required to cover this increase in volume. In this case, the PNS could be used to overcome the limitations of the lab space and the number of cameras needed." | Thank you for the suggestion.  "In addition, results from this study could potentially provide evidence for researchers that the PNS could be a viable tool to collect motion capture data in a more representative environment outside of a lab setting. This is especially so when it is not feasible to set up an optical based system. For example, in situations where there is a need to analyse movement over a larger area, if an optical based system is used, a greater number of cameras would be required to cover this increase in volume. In this case, the PNS could be used to overcome the limitations of the lab space and the number of cameras needed." This has been added to page 6-7, row 139 – 146. |
| Regarding the instrumentation section – I did not see a frame rate for the IMU system, did I miss it? | The frame rate for the IMU system was at 120Hz. It was stated on page 7, row 163. |
| Line 177 – the correct number of markers is 33, right? | Yes, it is 33. The amendment was made on page 8, row 168. |
| Data analysis - Since the specific algorithm you used is not completely clear, nor is the method used to synch the data, I would suggest publishing your code or else describing what was done here in more detail. It’s probably easier to just post the MATLAB code, I would think. | Thank you for the suggestion. The MATLAB code has been uploaded onto the NIE data repository and can be accessed through this link: https://doi.org/10.25340/R4/T6W5RX |
| The Sovitzy-Golay filter may not be as well known in the biomechanics community as you think. My recollection is that it is a moving average method involving convolution of low order polynomials. I would describe this more clearly, especially since there are questions about your filter frequency which I don't think specifically applies with this type of smoothing. Since different polynomials may be fit to different portions of the data, it seems that there is non-uniform smoothing which can match the local roughness of the data better and that the “frequency” is polynomical dependent. This is discussed in the white-paper found here:  [https://www.hpl.hp.com/techreports/2010/HPL-2010-109.pdf](about:blank)  Alternatively, I would cite a source describing these filters and their response, presuming that the other reviewer is content with this sort of explanation. | Thank you for the suggestion. The citation for the MATLAB code has been added on page 10, row 207. More information about the Savitzky-Golay filter has been included in the revised submission on page 10, row 205 – 208. |
| Table 3: Generally, correlations are done between series of data. In this case, I would expect a correlation calculated between VICON and IMU data per joint per activity and for each subject. The cited SD is then the SD of correlation coefficients across all subjects, right?  Each correlation test has a p-value. What is the p-value shown here if multiple correlation tests were done to determine the mean correlation and its standard deviation? Please clarify. | Sorry for the confusion. As you have mentioned a correlation was calculated per joint and per activity. The standard deviation, mean and p-value shown was based on the average of these correlations. Further clarifications were included in the revised submission on page 11, row 215 – 217. |
| Finally – I did not find your data in the NIE Data Repository – please post a URL! | Apologies for this. The data can be accessed through this link: https://doi.org/10.25340/R4/T6W5RX |
