# Supplementary material for: Validation of the Perception Neuron system for full-body motion capture
Source: PLoS One. 2022 Jan 21;17(1):e0262730. doi: 10.1371/journal.pone.0262730 (PMC8782534; doi:10.1371/journal.pone.0262730)
Supplement: Attachment — Submitted filename: Responses to Reviewers.docx [file pone.0262730.s005.docx]

**Responses to Reviewers**

Thank you for the points raised. Below, we highlight the revisions that have been made.

| **Reviewer 2 comments** |  |
| --- | --- |
| Thank you for the updates. However, your DOI (https://doi.org/10.25340/R4/T6W5RX) is returning a "DOI not found" from doi.org. Please check this and ensure that it is posted correctly in your final draft so that readers of the paper may access this information. This is my sole reason for selecting "Minor Revision" rather than "Accept" at this point. | Apologies for that. The data can be accessed through this updated link: <https://doi.org/10.25340/R4/IZKYZR>  However, the link would only be active when the paper is published.  This has been updated in the submission steps as well, under the section ‘Describe where the data may be found in full sentences. If you are copying our sample text, replace any instances of XXX with the appropriate details.’. |

| **Academic Reviewer comments** |  |
| --- | --- |
| Please add the description about the compression garments in the manuscript, which you made in the response to the reviewer 1 with the citation of Mills's paper. | The description about the compression garments have been added into the Manuscript on page 9, line 194-198. |
